# Dual-Functional Lithiophilic/Sulfiphilic Binary-Metal Selenide Quantum Dots Toward High-Performance Li–S Full Batteries

**DOI:** 10.1007/s40820-023-01037-1

**Published:** 2023-03-15

**Authors:** Youzhang Huang, Liang Lin, Yinggan Zhang, Lie Liu, Baisheng Sa, Jie Lin, Laisen Wang, Dong-Liang Peng, Qingshui Xie

**Affiliations:** 1 State Key Lab for Physical Chemistry of Solid Surfaces, Fujian Key Laboratory of Surface and Interface Engineering for High Performance Materials, College of Materials, Xiamen University, Xiamen, 361005 People’s Republic of China; 2 Shenzhen Research Institute of Xiamen University, Shenzhen, 518000 People’s Republic of China; 3 College of Materials Science and Engineering, Multiscale Computational Materials Facility, Fuzhou University, Fuzhou, 350100 People’s Republic of China

**Keywords:** Dual-functional host, Fe_2_CoSe_4_ quantum dots, Shuttle effect, Dendrite-free Li anode, Li–S full batteries

## Abstract

The commercial viability of lithium–sulfur batteries is still challenged by the notorious lithium polysulfides (LiPSs) shuttle effect on the sulfur cathode and uncontrollable Li dendrites growth on the Li anode. Herein, a bi-service host with Co-Fe binary-metal selenide quantum dots embedded in three-dimensional inverse opal structured nitrogen-doped carbon skeleton (3DIO FCSe-QDs@NC) is elaborately designed for both sulfur cathode and Li metal anode. The highly dispersed FCSe-QDs with superb adsorptive-catalytic properties can effectively immobilize the soluble LiPSs and improve diffusion-conversion kinetics to mitigate the polysulfide-shutting behaviors. Simultaneously, the 3D-ordered porous networks integrated with abundant lithophilic sites can accomplish uniform Li deposition and homogeneous Li-ion flux for suppressing the growth of dendrites. Taking advantage of these merits, the assembled Li–S full batteries with 3DIO FCSe-QDs@NC host exhibit excellent rate performance and stable cycling ability (a low decay rate of 0.014% over 2,000 cycles at 2C). Remarkably, a promising areal capacity of 8.41 mAh cm^−2^ can be achieved at the sulfur loading up to 8.50 mg cm^−2^ with an ultra-low electrolyte/sulfur ratio of 4.1 μL mg^−1^. This work paves the bi-serve host design from systematic experimental and theoretical analysis, which provides a viable avenue to solve the challenges of both sulfur and Li electrodes for practical Li–S full batteries.

**Supplementary Information:**

The online version contains supplementary material available at 10.1007/s40820-023-01037-1.

## Highlights

- A bi-service host with lithiophilic/sulfiphilic Fe_2_CoSe_4_ quantum dots embedded in three-dimensional ordered nitrogen-doped carbon skeleton is elaborately developed for both the sulfur cathode and Li anode synchronously.
- The highly dispersed Fe_2_CoSe_4_ quantum dots can not only act as a redox accelerator to promote the bidirectional conversion of LiPSs but also regulate the uniform Li plating/stripping to mitigate the growth of Li dendrite.
- The assembled Li-S full batteries achieve excellent long-term cyclability and a remarkable areal capacity of 8.41 mAh cm^2^ at high sulfur loading of 8.50 mg cm^2^, and the pouch full battery also displays high capacity and cycling-stability at lean electrolyte condition.

**Supplementary Information:**

The online version contains supplementary material available at 10.1007/s40820-023-01037-1.

## Graphical abstract

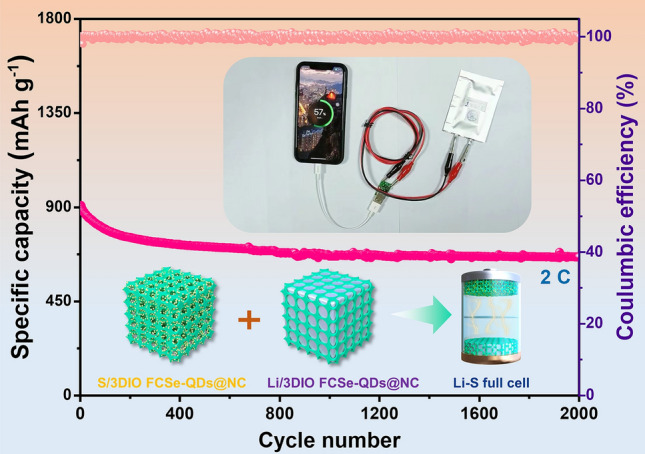

## Introduction

The surge of decarbonization has driven the rapid development of energy-storage technologies to meet the ever-expanding market and high-energy-demanding applications. Lithium-sulfur (Li–S) batteries are regarded as one of the most promising next-generation energy-storage systems owing to their high theoretical energy density and cost-effectiveness [1, 2]. Nevertheless, the commercialization of Li–S batteries is still challenged by various intractable obstacles in both sulfur cathode and Li anode. The so-called shuttle effect triggered by the dissolution of intermediate polysulfides (Li_2_S_*x*_, 4 ≤ *x* ≤ 8) leads to sluggish sulfur redox kinetics and unavoidable sulfur loss [3, 4]. Additionally, the infinite volume expansion and uncontrollable dendrite growth on the anode side result in low Coulombic efficiency (CE), electrical short circuits, and even security risk. Moreover, the commonly used Li foil anodes have a high negative to positive electrode capacity (N/P) ratio of over 50, much higher than practical relevance applications (< 10), sacrificing the superiority of the high-energy–density of Li–S batteries.

In light of these challenges, tremendous efforts have been dedicated to developing advanced host matrices for constructing high-efficiency sulfur cathode and Li-metal anode. At the cathode side, porous carbon materials with high electrical conductivity and large specific surface area were initially employed to physically confine lithium polysulfides (LiPSs) [5]. Nevertheless, the nonpolar carbon-based materials are inadequate in suppressing the shuttle effect due to the weak affinity for polysulfides [6]. Therefore, a synergetic strategy of combining conductive carbon matrices and polar materials (such as various heteroatoms [7], metal compounds [8–11], and alloys [12, 13]) is reasonably brought forward. But unfortunately, in these composite materials, some of the polar components (e.g., transition metal oxides and sulfides) generally exhibit such poor electrical/ionic conductivity that sluggish sulfur kinetics during redox reactions, which causes serious challenges in attaining high areal capacity Li–S batteries [14]. Alternatively, transition metal selenides (TMSes) have attracted immense attention because of their high electronic conductivity and unique electronic structure for appropriate adsorption and catalytic ability [15, 16]. And various selenides such as CoSe [15], ZnSe [17], MoSe_2_ [18], NbSe_2_ [19] have been frequently explored as catalytic additives for efficient sulfur hosts to improve the performance of Li–S batteries. Nevertheless, it should be noted that single-component TMSes usually suffer from relatively low electronic conductive properties and unsatisfied chemical stability, resulting in worse redox LiPSs kinetics and suboptimal stability during long-term cycling [20]. Instead, binary-metal TMSes with a rich electronic reaction process and improved electrochemical activity possess a strong polar surface to chemically adsorb LiPSs and can provide more efficient electrons to LiPSs, that is, stable, fast charge transport and durable catalytic effects can be achieved through multicomponent synergy and electronic band structure regulation [20], so the binary-metal TMSes are considered to be more efficient polysulfide mediators for shuttle effect inhibition. On the other hand, to maximize the amount of LiPSs adsorption sites and catalytic activity, the TMSes additives should be nanostructured. Among nanomaterial engineerings, quantum dots (QDs) materials, with ultra-small size and excellent dispersibility, have been demonstrated can greatly strengthen the host–guest interactions and reduce the reaction energy barrier [21], and the unique quantum connection endows them with an excellent catalysis effect for LiPSs [22]. Benefiting from these characteristics, the metal oxide QDs [23], carbon QDs [21], and phosphorus QDs [24] have been widely studied and applied in Li–S batteries, while the exploration of TMSes-based QDs for sulfur electrochemistry improvement is rarely reported before.

In addition to the sulfur cathode, the Li reaction behaviors at the anode side should be also regulated since the low conversion efficiency and uncontrollable dendrites growth takes weighty responsibility for the electrochemical performance degradation [25]. To solve the Li anode problems, various inspiring strategies have been developed, including electrolyte composition optimization [26], interfacial engineering [27], and rational construction of Li host [28]. Considering that the Li deposition behavior is strongly associated with the current density distribution, 3D Li host architectures have been passionately investigated in light of their high specific surface area and large interior space [28]. Of which, 3D carbonous substrates such as graphene [29], carbon nanotube/fiber [30], and highly ordered pyrolytic graphite [31], which would react with Li metal to produce LiC_6_ with excellent lithiophilicity and ionic conductivity, have been widely developed to effectively stabilize Li anode. However, facing the same dilemma, carbon-structured frameworks are generally inadequate in lithiophilicity, which inevitably results in inhomogeneous Li deposition and Li dendrites growth during long-term cycling since electrochemical Li deposition suffers from limitations in spreading and infiltrating due to the lithiophobic property [31]. Additionally, such randomly assembled micro-/nanostructures of 3D hosts without ordered spatial architectures would further exacerbate the inhomogeneity of the electric field distribution and Li-ion flux, especially true at high current density [32]. To address such challenges faced by both sulfur cathode and Li anode, it is desirable to construct a host matrix integrated with abundant lithiophilic/sulfiphilic species and rational ordered spatial structure to accelerate sulfur redox kinetics and regulate Li deposition behaviors synchronously, and finally enhance the electrochemical performance of Li–S batteries, which is of great significance but still remain challenges.

Herein, a bi-service host with Co-Fe binary-metal selenide quantum dots embedded into a 3D-ordered inverse opal structured N-doped carbon skeleton (3DIO FCSe-QDs@NC) is developed for both sulfur cathode and Li anode simultaneously. Both density functional theory (DFT) calculations and comprehensive experimental analysis demonstrate that the FCSe-QDs show excellent lithiophilic and sulfiphilic features, which act as both sulfur redox accelerator and Li deposition regulator for highly effective Li–S electrochemistry. As a sulfur host, the highly dispersed FCSe-QDs with strong chemisorption capability and superior catalytic activity can effectively promote the anchoring and conversion of LiPSs. As an anode host, the ordered open networks enable the homogeneous electric field distribution and lithiophilic FCSe-QDs react with Li to form metallic Co, Fe, and corresponding Li_2_Se seed layer. Such initially formed interphase ensures the uniform current distribution and Li-ion flux, thus regulating the subsequent Li deposition. Benefiting from these features, the assembled Li–S full batteries with 3DIO FCSe-QDs@NC host exhibit excellent electrochemical performance in terms of cycling stability and rate capability. Moreover, a promising areal capacity as high as 8.41 mAh cm^–2^ is achieved as the sulfur loading up to 8.5 mg cm^–2^ with a low N/P of 2.1:1, and the pouch full battery also displays high capacity (985 mAh g^–1^ at 0.3C) and excellent capacity retention even at lean electrolyte condition. These results collectively demonstrate the great potential of dual functional 3DIO FCSe-QDs@NC host for the practical application of Li–S batteries.

## Experimental Section

### Synthesis of Co-Fe Binary-Metal Oleate

Briefly, 5 mmol of CoCl_2_·6H_2_O, 5 mmol of FeCl_3_·6H_2_O, and 30 mmol of sodium oleate were successively dissolved in the mixture solvent composed of 20 mL of ethanol, 20 mL of distilled water, and 35 mL of hexane. Then, the resulting mixture was refluxed at 80 °C for 5 h under continuous stirring. When the reaction was completed and the mixture solvent was cooled down to room temperature. The upper organic layer containing Co-Fe oleate was washed with distilled water for several times to remove the NaCl composition. After standing for a period of time, the transparent aqueous solution at the bottom was completely separated, and the dark black upper oleate complex was obtained.

### Synthesis of 3DIO FCSe-QDs@NC, FCSe-QDs@NC, and 3DIO NC Composites

Firstly, silica nanospheres were prepared from modified Stöber’s method [33]. 0.5 g of the silica nanospheres, 1.0 g of Co-Fe-oleate complex, and 0.5 g of selenium powder were mixed completely by wet grinding with the induction of 1 mL of oleylamine. After being mixed thoroughly, the mixture was transferred to an alumina boat for thermal processing under an argon/hydrogen atmosphere with a heating rate of 5 °C min^−1^ to 650 °C and maintained for 3 h. Finally, the resulting black powder was dissolved in a 2 M NaOH solution and stirred overnight to remove the silica templates, and the 3DIO FCSe-QDs@NC was obtained. For comparison, the synthesis of FCSe-QDs@NC was similar to 3DIO FCSe-QDs@NC except for the absence of silica templates. The 3DIO NC was prepared by calcining the mixture of the cobalt-based oleate and silica nanospheres and removing the attached active sites through acid treatment, remaining the IO structure nitrogen-doped carbon matrices.

### Fabrication of S/3DIO FCSe-QDs@NC, S/FCSe-QDs@NC, and S/3DIO NC Cathodes

The sulfur cathodes were prepared by the classical melting diffusion method. The host matrices and sublimed sulfur with a weight ratio of 3:7 were mixed through grinding in a mortar. Then, the mixture was transferred into the reaction kettle and heated at 155 °C for 12 h to obtain the sulfur composite. The obtained sulfur composite was mixed with acetylene black and polyvinylidene difluoride (PVDF) at a weight ratio of 8:1:1 in N-methyl-pyrrolidinone (NMP) to form a homogeneous slurry, followed by blading coating onto the aluminum current collector to prepare S/3DIO FCSe-QDs@NC (or S/FCSe-QDs@NC and S/3DIO NC) electrodes with a diameter of 12 mm. The loading mass of active sulfur on the aluminum current collector is about 1.5 ~ 2 mg cm^−2^.

More details of other syntheses and characterizations can be seen in Supporting Information.

## Results and Discussion

### Fabrication and Characterization of 3DIO FCSe-QDs@NC

**Fig. 1:**
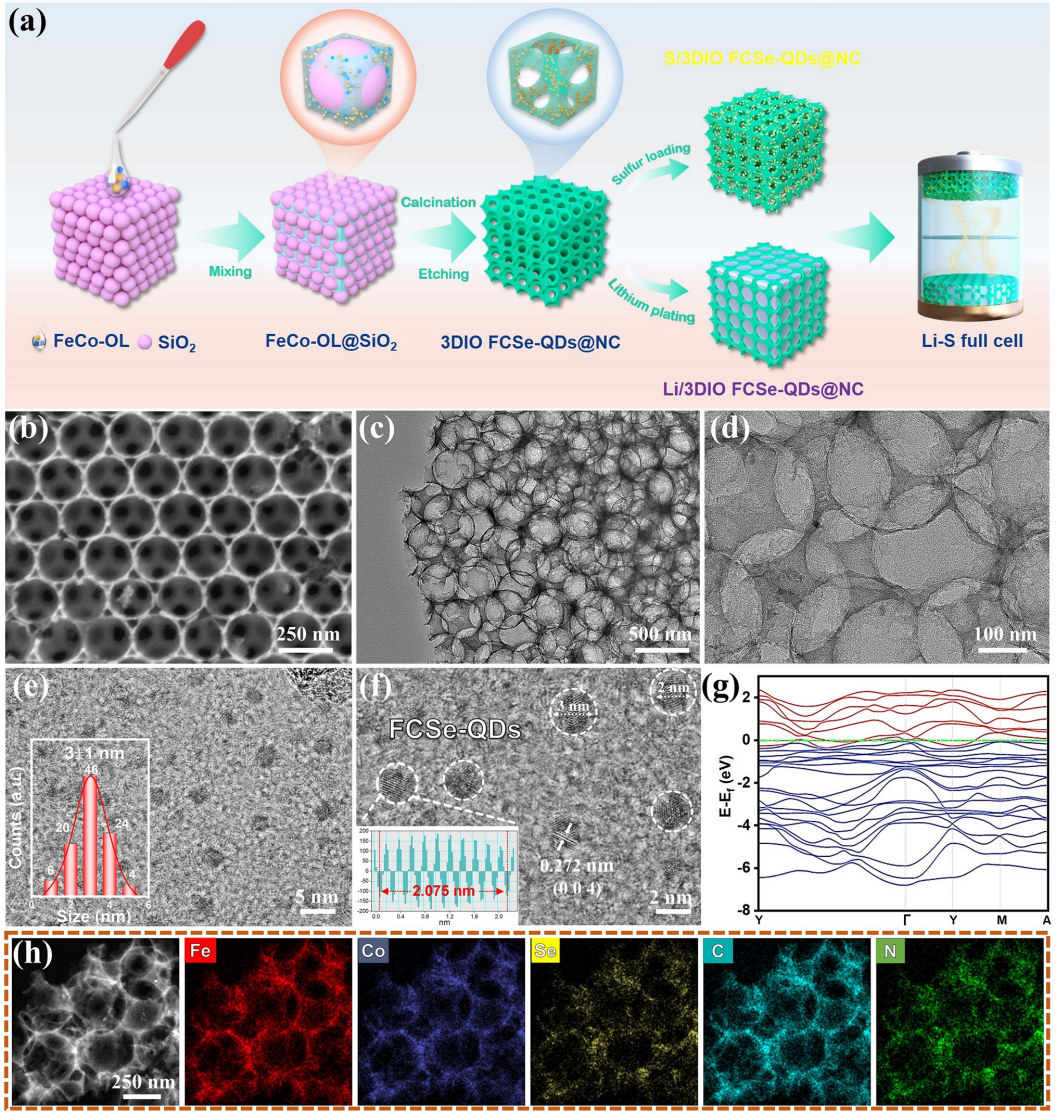
**a** Schematic illustration for the preparation of 3DIO FCSe-QDs@NC and its application as a two-in-one host for both sulfur cathode and Li metal anode. **b** SEM images, **c, d** TEM images, and **e, f** HR-TEM images of 3DIO FCSe-QDs@NC. **g** Energy band structure of the Fe_2_CoSe_4_ crystal, **h** SEM image and corresponding elemental mappings of 3DIO FCSe-QDs@NC

The synthetic procedure of the 3DIO FCSe-QDs@NC is schematically illustrated in Fig. 1a. At first, the Co-Fe binary-metal oleate (Co-Fe OL) was obtained by reacting sodium oleate with a specific ratio of iron-cobalt chloride, and silica nanospheres were served as the 3D ordered matrix directing agent (Fig. S1). The as-prepared Co-Fe OL, selenium powder, and oleylamine were mixed completely through a wet grinding process. Then the mixture was calcined in an argon/hydrogen atmosphere, during which the capping ligands of oleylamine and oleate were converted into the homogeneous nitrogen-doped carbon skeleton, and metal ions reacted with the hydrogen selenide atmosphere (H_2_ + Se → H_2_Se) to in situ form the Fe_2_CoSe_4_ QDs (FCSe-QDs). Finally, the 3DIO architecture carbon skeleton embedded with highly dispersed FCSe-QDs was obtained after removing the silica template.

Figures 1b and S2 display the typical scanning electron microscopy (SEM) images of 3DIO FCSe-QDs@NC at different resolutions, in which the 3D cross-linked porous morphology is observed clearly, and holes are arranged in a regular inverse opal structure with no particle agglomeration on the surface. Transmission electron microscopy (TEM) images further verify the hollow-ordered macroporous structure with cross-linked nanocages and the pore size is about 220 nm (Fig. 1c, d). This unique morphology would attribute to the strong binding affinity between metal-oleate molecules and silica surfaces. Such interconnected porous architecture can not only ensure sufficient sulfur loading and expose interface active sites for surface electrochemical reactions, but also can serve as conductive scaffolds for expediting electron transfer. HRTEM images in Fig. 1e suggest that the punctate FCSe-QDs are homogeneously implanted into the carbon skeleton with an average diameter size of about 3 nm, and the lattice fringes of 0.207 and 0.272 nm are assigned to the (-202) and (004) lattice planes of the Fe_2_CoSe_4_ phase, respectively (Figs. 1f and S2).

X-ray diffraction (XRD) pattern shows that all the diffraction peaks are well indexed to the Fe_2_CoSe_4_ phase (JCPDS No. 89–1967) after removing the SiO_2_ templates, demonstrating the successful evolution of the metal-oleate in the hydrogen selenide atmosphere (Fig. S2a). Thermogravimetric analysis (TGA) was performed in the air to estimate the weight ratio of Fe_2_CoSe_4_ in 3DIO FCSe-QDs@NC and the large weight decrease in the range from 600 to 800 °C is related to the conversion of the selenide into oxides (Fig. S3). Therefore, the weight ratio of Fe_2_CoSe_4_ is calculated to be about 81 wt%. Moreover, the heteroatom nitrogen-doping can serve as a Lewis-base site to interact with the Lewis acid site of LiPSs, which is favorable for LiPSs immobilization (Fig. S4). Additionally, it is worth mentioning that the phase composition is closely related to the feed ratio, when the Fe-OL alone was utilized as the starting precursor, the pure iron selenide (JCPDS No. 75–0608) phase was obtained (Fig. S5). By introducing a moderate amount of Co precursor (Co: Fe = 1:2), in addition to the diffraction peaks of Fe_2_CoSe_4_, the diffraction peaks of selenide cobalt (CoSe_2_: JCPDS No. 53–0449 and No. 09–0234) appear. As increasing the concentration of Co to 100%, the final product contains only the CoSe_2_ phase. These results evidence the success of our proposed strategy to produce binary-metal selenide QDs in a simplistic way. Figure 1h shows the energy-dispersive X-ray spectroscopy (EDS) elemental mappings of 3DIO FCSe-QDs@NC, wherein C, Fe, Co, Se, and N elements are distributed homogeneously, certifying the good dispersion of FCSe-QD, and successful doping of nitrogen in the inverse opal carbon frameworks, which are helpful for the LiPSs immobilization [6]. In addition, the electronic structure is presented to gain insight into the catalytic properties of 3DIO FCSe-QDs@NC. As shown in Fig. 1g, peaks corresponding to the calculated density of states of FCSe/NC are absent near the Fermi energy, demonstrating that the decoration of FCSe with metallic-like property can greatly improve the intrinsic conductivity of 3DIO carbon matrices and favor electron transfer during redox reactions. For comparison, the bulk FCSe-QDs@NC and 3DIO NC without FCSe-QDs loading were also synthesized as reference samples (Figs. S6 and S7). Note that the QDs of FCSe-QDs@NC are embedded in the interior and densely packed, which might not conducive to the exposure and full utilization of active sites.

Figure S8 displays the Raman spectra of the prepared host matrix, in which two strong Raman peaks at 1338 and 1590 cm^–1^ are assigned to the *sp*^3^-type disordered carbon form (D band) and *sp*^2^-type graphitized carbon form (G band), respectively. The 3DIO FCSe-QDs@NC shows a higher G/D ratio than that of FCSe-QDs@NC and FCSe-QDs@NC, indicating its higher graphitization degree in the carbon layer. Besides, the 3DIO FCSe-QDs@NC exhibits a high Brunauer–Emmett–Teller (BET) surface area and pore volume of 326 m^2^ g^–1^ and 0.441 cm^3^ g^–1^, respectively (Fig. S9). From the pore size distribution, the hierarchical porous structure consists of micropores, mesopores, and macropores, and pore size is mainly concentrated at 1 ~ 10 nm. Such a large specific surface area and hierarchical porous characteristics are favorable for sulfur loading and volume change mitigation upon cycling.

### Chemisorption and Electrocatalytic Effects of 3DIO FCSe-QDs@NC

**Fig. 2:**
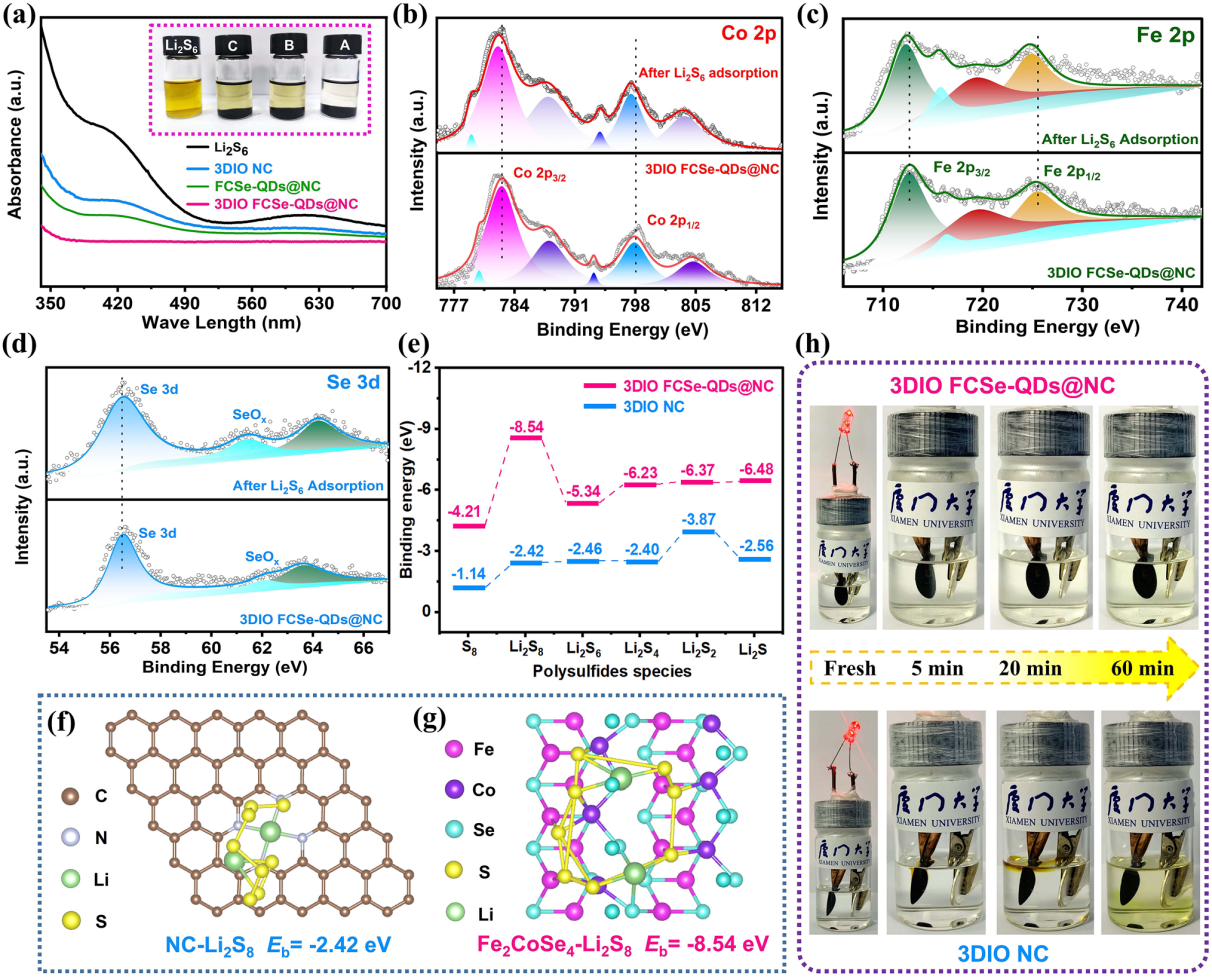
**a** Optical photograph and UV–vis spectra of Li_2_S_6_ solutions containing different adsorbents after resting for 6 h. High-resolution XPS spectra of **b** Co 2*p*, **c** Fe 2*p*, and **d** Se 3*d* of 3DIO FCSe-QDs@NC before and after Li_2_S_6_ adsorption. **e** Calculated binding energies of S_8_ and LiPSs (Li_2_S_8_, Li_2_S_6_, Li_2_S_4_, and Li_2_S_2_) and **f**, **g** corresponding optional binding structures of Li_2_S_8_ adsorped on 3DIO FCSe-QDs@NC and 3DIO NC surfaces. **h** Visual illustration of polysulfide entrapment at different discharge stages in bottle-assembled batteries of 3DIO FCSe-QDs@NC (*top*) and 3DIO NC (*bottom*)

On the cathode side, preventing the soluble polysulfides from diffusing is the first step to suppress the shuttle effect, thus the adsorption capability of host materials plays a vital role in immobilizing LiPSs. In this respect, the Li_2_S_6_ visualization adsorption tests were conducted to probe the chemical affinity toward with LiPSs. As observed from the inset optical images in Fig. 2a, the Li_2_S_6_ solution containing FCSe-QDs@NC and 3DIO NC adsorbent shows light yellow color after soaking for 6 h, while the Li_2_S_6_ solution mixed with 3DIO FCSe-QDs@NC is fully decolored, intuitively testifying the stronger affinity for polysulfides. The adsorption effects are also confirmed by UV–vis spectra, in which 3DIO FCSe-QDs@NC shows the weakest polysulfides characteristic peaks, corresponding to the hardly amount of polysulfides ions in the solution and thus the greatest LiPS adsorbability. Besides, according to the X-ray photoelectron spectroscopy (XPS) analyses in Fig. 2b, c, both Co 2*p* and Fe 2*p* peaks of 3DIO FCSe-QDs@NC-Li_2_S_6_ shift to lower binding energies than those of pristine 3DIO FCSe-QDs@NC, confirming the increased electron density located at the metal center. Conversely, the Se 3*d* XPS spectrum of 3DIO FCSe-QDs@NC-Li_2_S_6_ shifts to higher binding energies, which indicates an increase of the chemical environment electronegativity during the LiPS adsorption. These observations convincingly verify the existence of obvious chemical interaction between 3DIO FCSe-QDs@NC-Li_2_S_6_ and LiPSs, viz. the strong anchoring ability of 3DIO FCSe-QDs@NC-Li_2_S_6_ for LiPSs [23, 34].

Density functional theory (DFT) calculations were performed to investigate the intrinsic interfacial interaction mechanism between adsorbent and LiPSs at the atomic level. The binding energies and atomic structures between LiPSs and FCSe and bare NC were calculated. As summarized in Fig. 2e, the FCSe possesses higher binding energies (*E*_b_) at all lithiation stages than those on bare NC. Especially, the FCSe delivers an extremely high *E*_b_ of -8.54 eV for Li_2_S_8_ on the (001) lattice plane, which is more than three times that on unadorned NC (-2.42 eV) (Fig. 2f, g). Additionally, Figs. S10 and S11 display the optimized LiPSs adsorption configuration at different lithiation stages (Li_2_S, Li_2_S_2_, Li_2_S_4_, Li_2_S_6_, Li_2_S_8_, and S_8_). The coexistence of multiple adsorption sites to strongly capture the LiPSs by Fe/Co-S and Li-Se bonds endow the great potential of 3DIO FCSe-QDs@NC composite as high-efficiency sulfur host [35]. And, the strong adsorbability is favorable for the smooth progress of subsequent complex electrocatalytic reactions on the 3DIO FCSe-QDs@NC electrode [36].

Sulfur was infiltrated into 3DIO FCSe-QDs@NC nanoarchitecture by a typical melt-diffusion method, and the loaded mass of sulfur is about 70.2 wt% from thermogravimetry analysis (Fig. S12a). Notably, the 3D-ordered porous structure is well maintained after sulfur loading and EDS elemental mappings display homogeneous distribution of sulfur with no apparent particle agglomeration (Fig. S12b-i). To observe the dissolution and diffusion behavior of soluble LiPSs in real time, the optically transparent bottle cells were assembled with different cathode and Li foil anode in a clear electrolyte and connected to an external light bulb to discharge. As exhibited in Fig. 2h, the transparent electrolyte quickly turned to yellow for the S/FCSe-QDs@NC and S/3DIO NC electrode (less than 30 min), owing to the overflow of LiPSs from the electrode surface and diffuse into the electrolyte. In stark contrast, one can clearly observe that the electrolyte color of the S/3DIO FCSe-QDs@NC-based bottle cell remains transparent during the whole discharge process even lasting for 60 min, indicating the strong immobilization ability toward LiPSs owing to the customized porous structure and abundant adsorption sites [37].

**Fig. 3:**
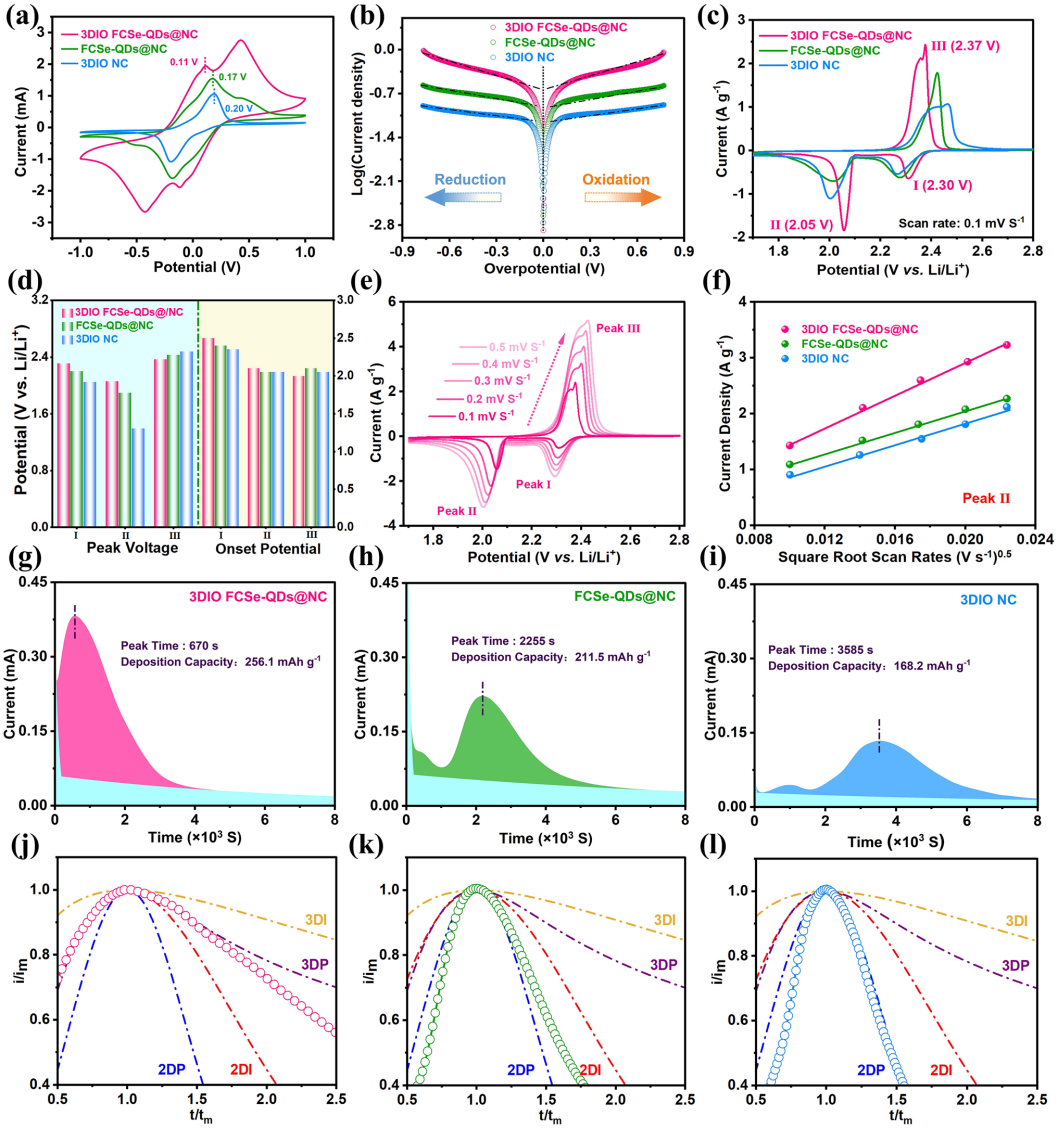
**a** CV curves of symmetric cells assembled with 3DIO FCSe-QDs@NC, FCSe-QDs@NC, and 3DIO NC electrodes at 5 mV s^−1^. **b** Tafel plots of symmetric cells. **c** CV curves of different Li–S cells at 0.1 mV s^–1^. **d** The corresponding peak voltages and onset potentials from CV curves. **e** CV curves tested at different scanning rates of 3DIO FCSe-QDs@NC and **f** peak current for the second electrochemical processes (II: Li_2_S_x_ → Li_2_S_2_/Li_2_S) *versus* the square root of the scan rates. **g–i** Li_2_S nucleation tests based on different electrodes for evaluating the nucleation kinetics. **j–l** Dimensionless current–time transient to perform peak fitting according to theoretical 2D and 3D models, *I*_m_ (peak current) and *t*_m_ (time needed to achieve the peak current) detected from the current–time transients

To evaluate the ability of host materials to catalytically accelerate the kinetics of polysulfide conversion, cyclic voltammetry (CV) profiles of Li_2_S_6_ symmetrical cells were collected in the voltage window of –1.0 to 1.0 V (Fig. 3a). All the symmetrical cells exhibit two pairs of redox peaks, corresponding to the conversion of S_8_ to Li_2_S_6_ and then to Li_2_S, respectively, and vice versa [3]. Among them, the 3DIO FCSe-QDs@NC harvests the sharpest redox peaks and smallest voltage polarization compared with other symmetric cells, indicative of accelerated redox kinetics and enhanced catalytic activity [38]. Notably, the 3DIO FCSe-QDs@NC-based symmetrical cell still maintains two obvious redox peaks as the sweep rate up to 50 mV s^−1^, implying rapid charge transfer and low electrochemical polarization, and electrochemical impedance spectroscopy (EIS) results collectively verify the lowest impedance for 3DIO FCSe-QDs@NC electrode (Fig. S14). Additionally, the enhanced reaction kinetics and electrocatalytic activity were further investigated by Tafel plots in Fig. 3b, wherein the uppermost response current is obtained for 3DIO FCSe-QDs@NC in both anodic and cathodic processes. And the highest exchange current density of 0.14 mA cm^−2^ *vs.* FCSe-QDs@NC (0.064 mA cm^−2^) and 3DIO NC (0.041 mA cm^−2^) further reveals the fast electron transfer on the electrode-LiPSs interface in 3DIO FCSe-QDs@NC electrode.

Figure 3c shows the CV curves obtained from S/3DIO FCSe-QDs@NC, S/FCSe-QDs@NC, and S/3DIO NC electrodes. All curves exhibit two well-defined cathodic peaks, which are associated with the conversion of active sulfur to long-chain LiPSs (Li_2_S_*x*_, 4  ≤ *x* ≤ 8, peak A) and the subsequent conversion to insoluble Li_2_S_2_/Li_2_S (peak B). In turn, the anodic peaks correspond to the reverse oxidation conversion from Li_2_S to sulfur [39]. As a definition to evaluate the catalytic ability toward LiPSs, onset potential is defined as the current of 10 μA cm^–2^ surpassing the baseline current (see details in Supporting Information, Fig. S16) [40]. Obviously, the as-assembled cell based on S/3DIO FCSe-QDs@NC delivers the highest onset potential in cathodic peaks (both in Peaks A and B) and the lowest onset potential in anodic peaks (Peak C), testifying the accelerated reaction kinetics and dramatically decreased polarization during multi-step phase conversion (Fig. 3d). Figures 3e and S17 show the CV curves of three electrodes at different scanning rates, in which the S/3DIO FCSe-QDs@NC electrode still exhibits the lowest polarization and highest redox peak current at all scanning rates. Both cathodic and anodic peak currents (*I*_p_) show a linear dependence on the square root of the scanning rate (*v*^0.5^), which points to a diffusion-limited process [41]. According to the Randles–Sevcik equation (see Supporting Information for details), the Li^+^ diffusion coefficient (D_Li_^+^) at each step is positively correlated with the slope of the line [42]. Furthermore, the calculated ${D}_{{\mathrm{Li}}^{+}}$ for S/3DIO FCSe-QDs@NC at peaks I, II, and III are 2.15, 3.92, and 5.26 7 ×  10^−7^ cm^2^ s^−1^, respectively, which are also much higher than those of S/FCSe-QDs@NC and S/3DIO NC electrodes (as summarized in Table S1), illustrating its significantly facilitated Li^+^ diffusivity [43]. For Li–S batteries, it should be noted that the Li^+^ ion diffusivity strongly associates with the accumulation of the viscosity of the electrolyte that varies with the concentration of soluble LiPSs [44]. In this case, the highest Li^+^ ion diffusion rate of S/3DIO FCSe-QDs@NC electrode corroborates the improved chemisorption ability and catalytic activity of the embedded FCSe-QDs that can effectively immobilize LiPSs and prevent them from dissolving into the electrolyte.

It is recognized that a very significant part of the discharge capacity (~ 75%) stems from the conversion of Li_2_S_4_ into Li_2_S in the second stage, potentiostatic nucleation analysis was conducted to reveal Li_2_S nucleation efficiency (Fig. 3g-i). According to Faraday’s law, the nucleation capacity of the 3DIO FCSe-QDs@NC electrode is calculated to be 256.1 mAh g^–1^, well above that of FCSe-QDs@NC (211.5 mAh g^–1^) and 3DIO NC (168.2 mAh g^–1^) [45]. Furthermore, the response of Li_2_S nucleation on the 3DIO FCSe-QDs@NC-based cell is the earliest one, which indicates that the FCSe-QDs possesses good electrocatalytic activity toward LiPSs and effectively speeds up the kinetics of Li_2_S redox in the sulfur electrochemistry. Besides, in contrast to the irregular and clumpy growth morphology for FCSe-QDs@NC and 3DIO NC, a flat and uniform topography of Li_2_S deposition is found on the surface of 3DIO FCSe-QDs@NC (Figs. S18-S20, see details in the Supporting Information). Furthermore, to probe the regulation effects of FCSe-QDs toward Li_2_S distribution, the dimensionless current–time transient curves from potentiostatic discharge results are recorded and fitted with the four classical models of electrochemical deposition [46, 47]. 2D instantaneous nucleation (2DI) and 2D progressive nucleation (2DP) are based on Bewick, Fleischmann, and Thirsk models, both of which involve 2D nucleation with adatoms incorporated into the lattice [48]. 3D instantaneous nucleation (3DI) and 3D progressive nucleation (3DP) are founded on Scharifker-Hills models and represent the 3D hemispherical nucleus with an ion diffusion-controlled growth [47]. As exhibited in Fig. 3j, the Li_2_S precipitation process on the FCSe-QDs@NC and 3DIO NC electrodes strictly obeys a 2DI nucleation model (Fig. 3k, l). Instead, the 3DIO FCSe-QDs@NC electrode follows the 3DP model, indicating that its Li_2_S nucleation is not affected by the crystal plane, and it is easier to nucleate and grow. These results are fairly consistent with the SEM morphology analysis, which further evidenced the synergistic effect toward Li_2_S nucleation when combining host structure regulation and sulfiphilic sites introduction. In turn, 3DIO FCSe-QDs@NC also delivers the largest decomposition capacity in the potentiostatic charging tests, revealing the favorable bidirectional catalytic ability in boosting the Li_2_S dissolution (Fig. S21).

**Fig. 4:**
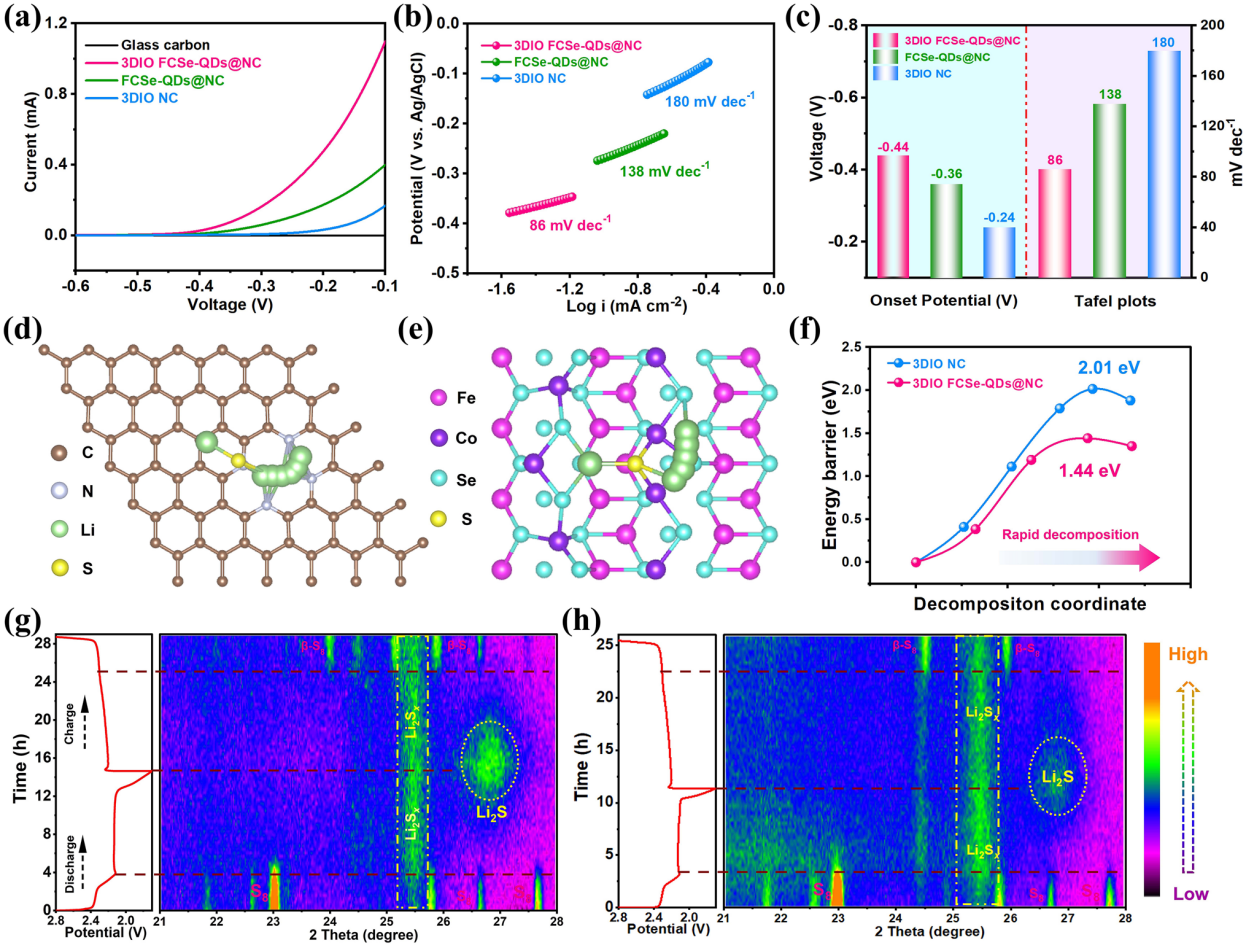
**a** LSV curves and **b** corresponding Tafel plots of Li_2_S oxidization on different substrates based on a three-electrode system. **c** Onset potentials and Tafel plot statistics from LSV tests. **d** Top view schematic representations of the decomposition pathways for 3DIO NC and **e** 3DIO FCSe-QDs@NC, and f) corresponding Li_2_S cluster decomposition energy profiles on different substrates. *In situ* XRD patterns in contour plots of the Li − S cells with **g** 3DIO FCSe-QDs@NC and **h** FCSe-QDs@NC electrodes during the first cycle

To reveal the advantage of 3DIO FCSe-QDs@NC toward polysulfide conversion in depth, the sulfide oxidation behavior on different hosts was studied by three-electrode linear sweep voltammetry (LSV), in which 0.1 M Li_2_S/methanol, a platinum sheet, and AgCl/Ag served as the electrolyte, counter, and reference electrodes, respectively. Obviously, compared with FCSe-QDs@NC (-0.36 V) and 3DIO NC (-0.24 V) electrodes, the 3DIO FCSe-QDs@NC possesses the lowest onset voltage (-0.44 V) as well as the largest current response, implying the lowest energy barrier from insoluble Li_2_S to soluble polysulfides and good catalytic ability. This conclusion is consistently supported by the Tafel plots in Fig. 4b, which deliver the smallest Tafel slope of 86 mV dec^−1^ for 3DIO FCSe-QDs@NC compared with those two electrodes. Furthermore, the bidirectional catalytic effect of 3DIO FCSe-QDs@NC is confirmed by the Li_2_S decomposition barrier calculated by the climbing-image nudged elastic band method (CI-NEB) (Fig. 4d-f). The Li evolution process is composed of the breaking of the Li–S bond and then moving far away. According to the calculation results, the decomposition barrier of Li_2_S on the FCSe surface is 1.44 eV, lower than that of 2.01 eV for bare NC, which points to an easier dissociation process of the Li_2_S under the regulation of the FCSe-QDs catalysis centers, thus the great enhancement of decomposition process. In addition, to directly monitor the polysulfide evolution and sulfur redox chemistry during the electrochemical process, *In situ* XRD tests were performed. As depicted in Fig. 4g, the initially detected characteristic peaks are assigned to the elemental orthorhombic *α*-S_8_ [49]. Then, the original sulfur peaks continuously decrease, and new, yet broad, long-chain polysulfide peaks around 25°-26° arise. Subsequently, the characteristic peak of crystalline cubic Li_2_S around 27.2° (JCPDS No. 23–0369) strengthens gradually and reaches its maximum at the fully discharged state [50]. During charging, the intensity of the Li_2_S peak decreases along with the generation of new peaks representing monoclinic *β*-sulfur (JCPDS No. 71–0137) at the end of final charged state. From the comparative results, it is easily found that the 3DIO FCSe-QDs@NC renders a much weaker polysulfide yield and exhibits a stronger and broader Li_2_S signal, while broad polysulfide signals for the other two electrodes (Fig. S22). These stark contrasts clearly reconfirm the greatly enhanced polysulfide-adsorption capability and redox-conversion reversibility contributed by 3DIO FCSe-QDs@NC.

**Fig. 5:**
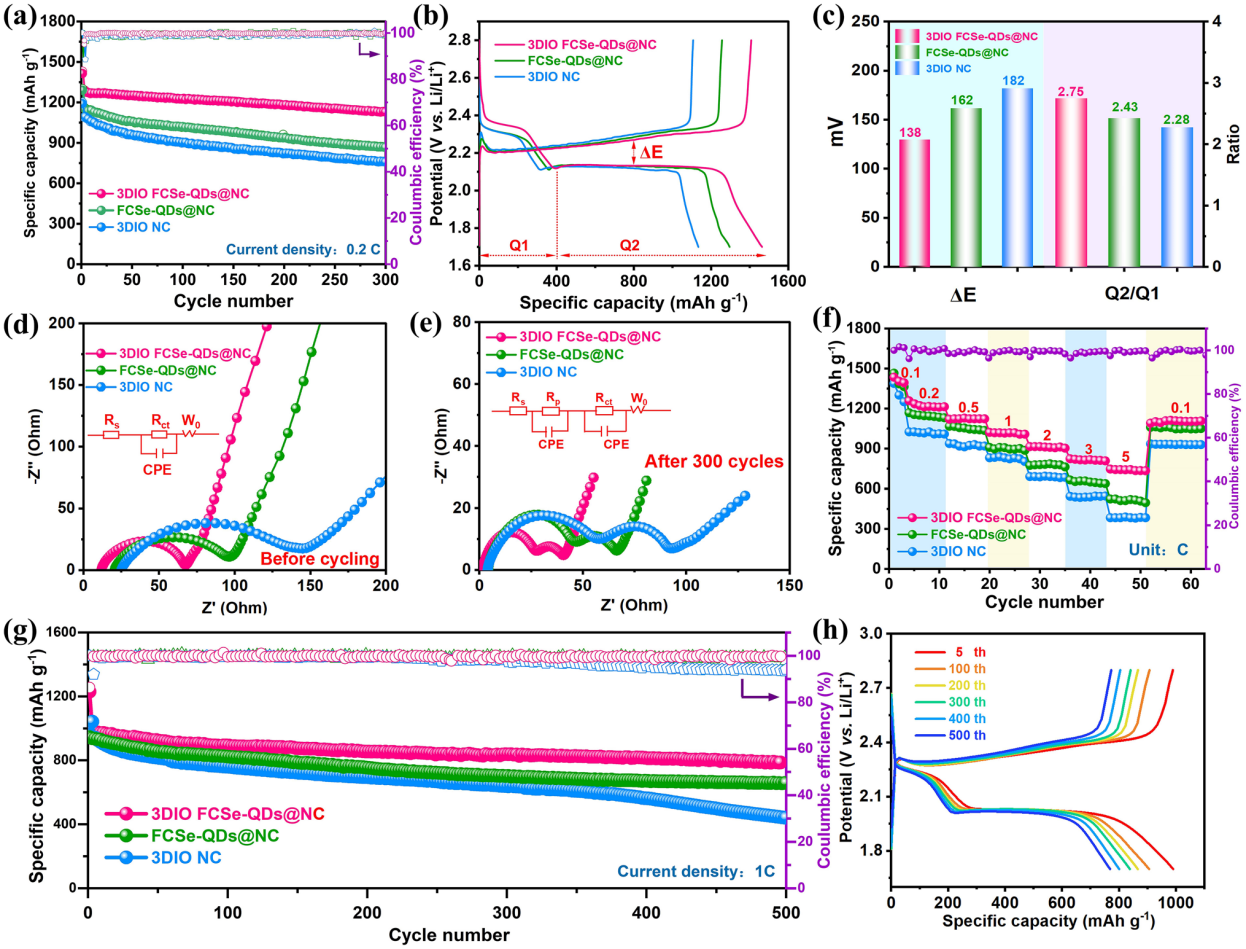
**a** Cycling performances and the corresponding coulombic efficiencies of the Li–S batteries with S/3DIO FCSe-QDs@NC, S/FCSe-QDs@NC, and S/3DIO NC electrodes at 0.2C. **b** Charge–discharge profiles of the cells at 0.2C. **c** Values of ΔE and η obtained from the charge–discharge profiles. **d, e** EIS spectra of the fresh electrodes and after 300 cycles at 0.1C. **f** Rate capability at current rates from 0.1 to 5 C. **g** Prolonged cycle life of the different sulfur cathodes at 1C and **h** charge–discharge profiles at different cycles

To investigate the difference in electrochemical performance based on different hosts, coin-type half-cells were constructed, of which, the S/3DIO FCSe-QDs@NC electrode delivers a significantly higher initial capacity of 1,406 mAh g^−1^ than 3DIO FCSe-QDs (1,340 mAh g^−1^) and 3DIO NC (1,210 mAh g^−1^) at 0.1C, and obtains a superior capacity retention rate of 82.3% after 300 cycles at 0.2C (Fig. 5a). Note that the pure host matrix without sulfur implantation is electrochemically inactive (Fig. S23). In terms of the galvanostatic charge/discharge (GCD) curves, the S/3DIO FCSe-QDs@NC electrode exhibits the smallest polarization potential of 138 mV among the three electrodes. And the ratio of capacities contributed from two discharge platforms (marked as ΔQ2/ΔQ1) can also be utilized to quantify the catalytic activity, where Q1 relates to the reduction of sulfur to soluble LiPSs and Q2 corresponds to the subsequent conversion transformation to Li_2_S, respectively [51]. The higher value of Q2/Q1, the better catalytic activity. As summarized in Fig. 5c, the S/3DIO FCSe-@NC delivers a higher Q2/Q1 ratio of 2.75 than that for S/FCSe-QDs@NC (2.43) and S/3DIO NC (2.28) electrodes, which is close to the theoretical value of 3, evidencing the enhance redox reaction kinetics in the conversion of Li_2_S_4_/Li_2_S_2_ to Li_2_S at the second stage.

Figure 5d, e shows the Nyquist plots of different cathodes before and after cycling. Compared with the initial EIS spectra, an additional semicircle in the high-frequency range is evidenced in the cycled cells, which is related to the interface resistance (*R*_p_) of the insoluble Li_2_S_2_/Li_2_S passivation layer [39]. Owing to the favorable interfacial kinetics, the 3DIO FCSe-QDs@NC electrode delivers the smallest internal resistance in both before and after cycling (Tables S2 and S3). Interestingly, the internal impedances are all decreased in the cycled cells, and the decrescent behaviors might ascribe to the full infiltration of electrolytes and the re-dispersion of sulfur species on the electrode surface [39]. As displayed in Fig. 5f, the S/3DIO FCSe-QDs@NC cathode fulfills good rate capability with high capacities of 1,223, 1,125, 1,221, 900, 830 and 781 mAh g^−1^ at 0.2, 0.5, 1, 2, 3 and 5 C, respectively, with clear plateaus at all current rates (Fig. S24). When reverses back to 0.2C, a high capacity of 1,098 mAh g^−1^ can be restored. By contrast, the S/FCSe-QDs@NC and S/3DIO NC cathodes undergo rapid capacity fading as the current rate increases. Furthermore, the S/3DIO FCSe-QDs@NC half-cell displays an excellent long-term cycling performance with a high reversible capacity retention of 801 mAh g^−1^ after 500 cycles at 1C (Fig. 5g), corresponding to a tiny capacity fading rate of 0.035% per cycle. The charge–discharge curves at different cycles further prove the electrochemical high stability and small polarization (Fig. 5h). In addition, to clarify the effect of different types of QDs on the electrochemical performance, the control samples with single metal QDs (3DIO CSe-QDs@NC and 3DIO FSe-QDs@NC) were also investigated. The adsorption tests and catalytic effect analysis are displayed in Fig. S25, in which single metal QDs hosts also exhibit comparable performance but all are slightly inferior to the binary-metal selenide, further verifying the superiorities of 3DIO FCSe-QDs@NC.

### Lithium Dendrite Growth Suppression by 3DIO FCSe-QDs@NC

**Fig. 6:**
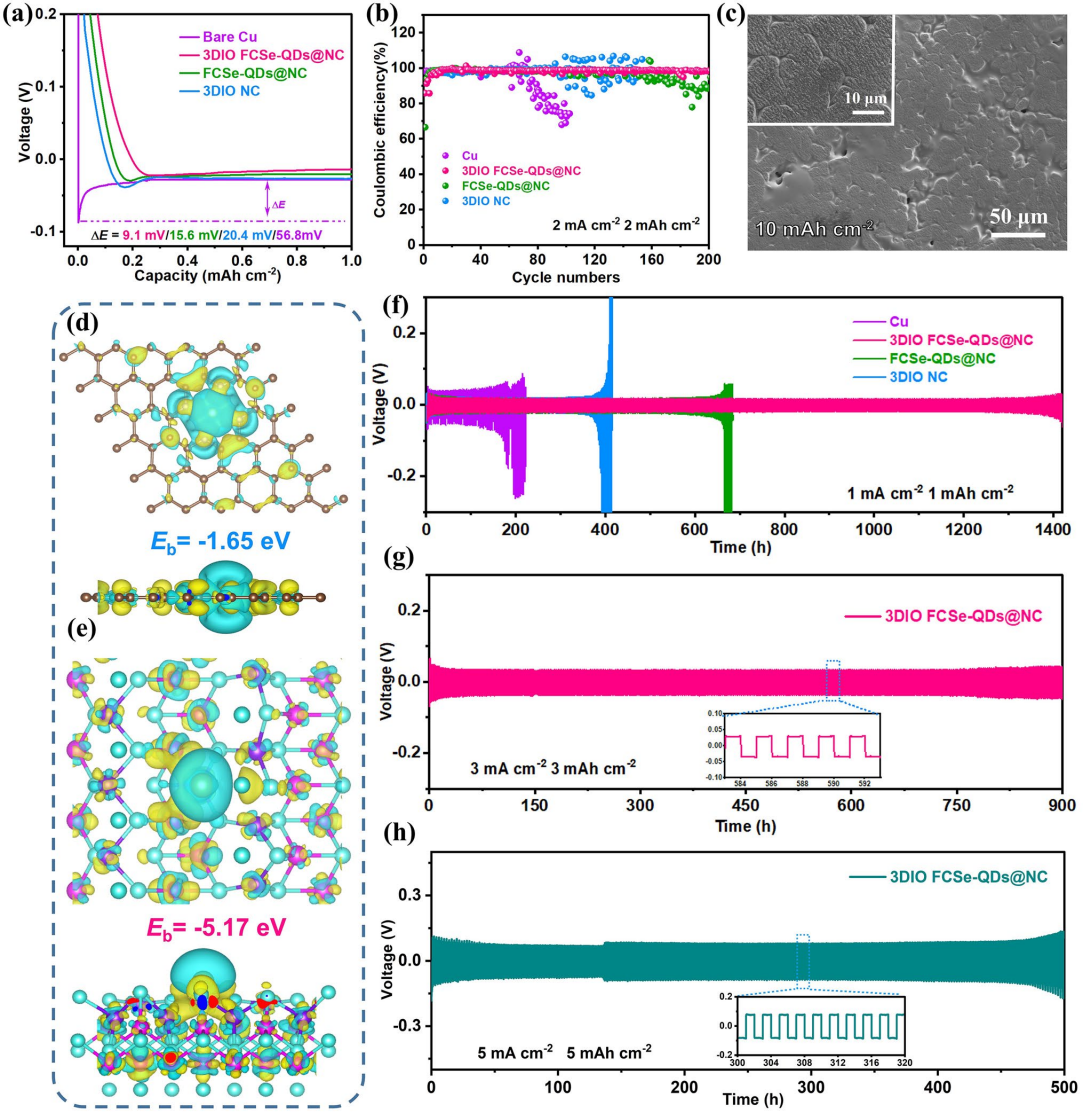
**a** The voltage–capacity profiles and **b** Coulombic efficiency of the Li plating-stripping progress on 3DIO FCSe-QDs@NC, FCSe-QDs@NC, 3DIO NC, and bare Cu foil hosts. **c** SEM images of Li deposition morphologies on 3DIO FCSe-QDs@NC host with a capacity of 10 mAh cm^−2^. **d, e** Calculated electron density differences of Li atom absorbed on 3DIO FCSe-QDs and 3DIO NC. **f** Galvanostatic cycling of symmetric cells based on Li/Cu, Li/3DIO FCSe-QDs@NC and Li/FCSe-QDs@NC, and Li/3DIO NC electrodes at 1 mA cm^−2^ for 1 mAh cm^−2^. Galvanostatic cycling of Li/3DIO FCSe-QDs@NC symmetric cells at **g** 3 mA cm^−2^ for 3 mAh cm^−2^ and **h** 5 mA cm^−2^ for 5 mAh cm^−2^

Given that the practical Li–S batteries equally suffer from the unacceptable growth of dendrite on the Li anode. The as-developed 3DIO FCSe-QDs@NC was further explored as a host matrix for the Li metal anode, in which the 3D-ordered porous structure integrated with the lithiophilic nature of FCSe-QDs ensures homogeneous Li^+^ flux and surface current filed, making 3DIO FCSe-QDs@NC as a promising candidate for Li dendrite inhibitor. CV curves in Fig. S26 clearly display multiple cathodic peaks arising from the formation of metal (Fe, Co) and Li_2_Se. These conductive mixed interphases have been proven to serve as mixed ion–electron conducting agents that can greatly enhance redox kinetics during Li plating [52]. Figure 6a compares the voltage profiles of Li metal plating onto different hosts, the 3DIO FCSe-QDs@NC host reveals the smallest nucleation overpotential of 9.1 mV *vs.* FCSe-QDs@NC (15.6 mV), 3DIO NC (20.4 mV) and bare Cu substrate (56.8 mV), indicating the excellent lithiophilicity of 3DIO FCSe-QDs@NC with reduced Li nucleation barrier. In the following cycling progress, the CE is collected to investigate the plating/stripping reversibility of the Li on the different hosts. As shown in Fig. 6b, in stark contrast to the violent fluctuations for FCSe-QDs@NC, 3DIO NC, and bare Cu electrodes, a stable Coulombic efficiency of > 99.1% is achieved for the 3DIO FCSe-QDs@NC within 200 cycles at 2 mA cm^–2^ and 2 mAh cm^−2^, certifying the significant synergistic effect of mixed ion–electron conductive interphase. Beyond that, the surface morphology evolution was investigated by *ex-*SEM to clarify the Li deposition behavior. The 3DIO FCSe-QDs@NC electrode exhibits an ultrafine and smooth Li nucleation morphology during the progressive plating progress, which is in vast contrast to the cavities and dendrites morphology on bulk FCSe-QDs@NC and Cu foil electrodes (Figs. S27-S30). Notably, even under an ultrahigh plating capacity of 10 mAh cm^–2^, a dense, smooth Li deposition layer with large grains of Li bulk is formed on the surface of 3DIO FCSe-QDs@NC electrode without obvious Li dendrites formation (Fig. 6c).

Based on the above results, to get insight into the Li adsorption ability. DFT calculations are carried out to determine the binding energies between Li atoms and different substrates. According to the calculation results in Fig. S31, the FCSe manifests a stronger interaction (−5.17 eV) than unadorned NC (-1.65 eV), indicating that FCSe is more lithiophilic and benefits the decrease of the nucleation overpotential of Li, which is consistent well with the aforementioned Li plating process. Moreover, the charge density difference was performed to understand the chemical origin of the above different binding energies (Fig. 6d, e), wherein the light blue and yellow colors indicate the regions of charge accumulation and depletion, respectively. Notably, there is strong electron density accumulation between Li and FCSe and reduced charge density around Li, implying that electrons are transferred from Li ions to neighboring FCSe atoms [53]. That is, Li ions tend to react with FCSe and prefer to nucleate around the QDs edges. Reversely, there is indeed an opposite display scene for unadorned NC.

Given that the plating/stripping stability of Li metal anodes plays a significant role in overall battery performance. Li symmetrical cells with different hosts were assembled and cycled with various current densities and capacities. As shown in Fig. 6f, the 3DIO FCSe-QDs@NC symmetric cell inherits the smallest overpotential of around 13 mV at 1 mA cm^–2^ and harvests impressive stability of more than 1,400 h with a small capacity of 1 mAh cm^–2^, while the other three cells behave in limited cycle lives with random voltage oscillations and increased polarization (Li/FCSe-QDs@NC: 685 h; Li/3DIO NC: 417 h; bare Cu: 256 h). Besides, Li/3DIO FCSe-QDs@NC electrode also delivers the lowest *R*_ct_ of 38.5 Ω, pointing to the formation of stable and high ion-conducing SEI film (Fig. S32 and Table S4). Admittedly, Li dendrites growth is usually caused by the large current density since the uniformity of Li^+^ ions flux strongly depends on the magnitude of current density [54, 55]. The electrochemistry stability of Li/3DIO FCSe-QDs@NC anode is further investigated under high current densities. As delivered in Fig. 6g, a long lifespan of over 800 h with a low voltage hysteresis of about 28 mV is obtained at 3 mA cm^−2^ and 3 mAh cm^−2^. More impressively, even rising to 5 mA cm^–2^ and 5 mAh cm^−2^, an outstanding Li stripping/plating stability is achieved with long-stable life of over 500 h, which is super to most of the state-of-the-art Li symmetrical cells reported previously (Table S5). Collectively, these satisfactory improvements testify that 3DIO FCSe-QDs@NC shows great potential for dendritic-free anodes by means of the unique 3DIO architecture and embedded lithiophilic FCSe-QDs.

### Performance of S/3DIO FCSe-QDs@NC||Li/3DIO FCSe-QDs@NC Full Batteries

**Fig. 7:**
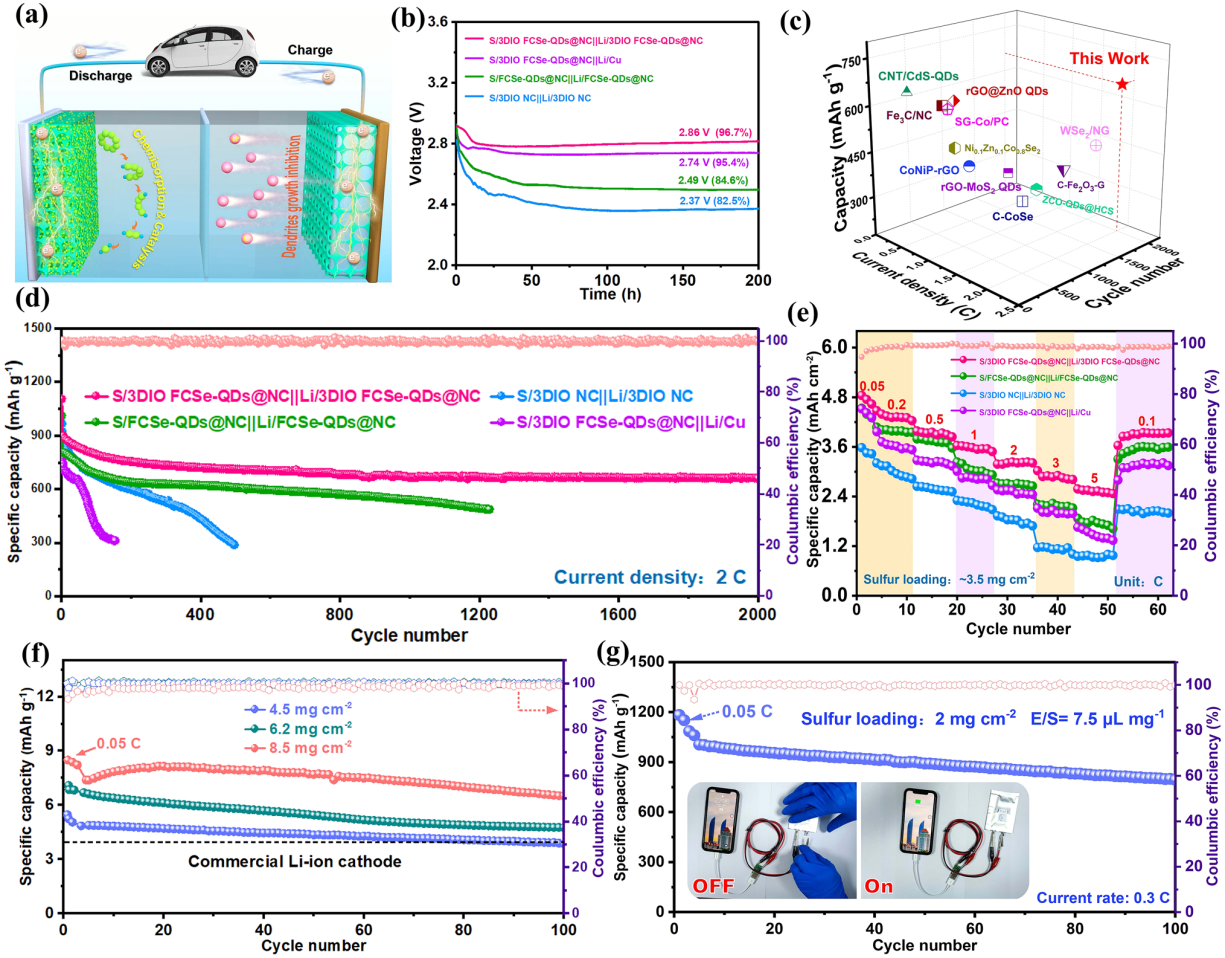
**a** Schematic illustration of the full cell constructed by S/3DIO FCSe-QDs@NC cathode and Li/3DIO FCSe-QDs@NC anode. **b** Time-dependent open circuit voltage evolution within 200 h. **c** Comparison of cycling performance of 3DIO FCSe-QDs@NC-based full cell with other previously reported Li–S batteries. **d** Prolonged cycle life of different assembled full cells. **e** Rate capability and **f** high sulfur loading measurements at 0.2 C. **g** Cycling stability of the pouch cell (size: 7 cm × 10 cm), inset shows the mobile phone charged by a pouch full cell

Encouraged by the excellent dual-functional properties of 3DIO FCSe-QDs@NC on both sulfur cathode and Li anode, the full cells coupling the S/3DIO FCSe-QDs@NC cathode and Li/3DIO FCSe-QDs@NC anode were established, as schematized in Fig. 7a. Since the self-discharge phenomenon caused by the shuttle effect is still a bottleneck for Li–S batteries, the self-discharge behaviors of the as-prepared full cells were firstly investigated by the open-circuit voltage profile (Fig. 7b). After resting for 200 h, the 3DIO FCSe-QDs@NC-based full cell exhibits an almost horizontal voltage curve with the highest voltage retention of 96.7% vs. FCSe-QDs@NC- (84.6%), 3DIO NC-based full cell (82.5%), and S/3DIO FCSe-QDs@NC||Li/Cu full cells (95.4%), revealing the effectively suppressed shuttle effect. Moreover, the long-term cycling performances are compared in Fig. 7d, an ultralow damping rate of 0.014% per cycle is harvested for 3DIO FCSe-QDs@NC-based full cell within 2,000 cycles at 2C, that is outperforming the state-of-the-art Li–S batteries (Fig. 7c and Table S6). Noticeably, the 3DIO FCSe-QDs@NC host from the disassembled cell displays the 3D ordered porous morphology without obvious structural degradation, indicating its excellent mechanical stability (see details in Fig. S33).

Nowadays, the pursuit of high-energy–density Li–S full batteries is strongly associated with high sulfur loading and low electrolyte dosage [56]. As depicted in Fig. 7f, the 3DIO FCSe-QDs@NC-based full cell presents an outstanding area rate performance with a superb discharge capacity of 4.56 mAh cm^–2^ at 0.2C under sulfur loading of about 3.5 mg cm^−2^, and a high reversible capacity of 2.61 mAh cm^−2^ is retained with the current rate up to 5C. And the galvanostatic charge/discharge profiles display distinct discharge plateaus even at high rates (Fig. S34), demonstrating the fast Li electrochemistry kinetics and sulfur conversion. Additionally, as the sulfur loading increases to 4.5 mg cm^–2^, the S/3DIO FCSe-QDs@NC-based full cell maintains a high areal capacity of 3.91 mAh cm^−2^ after 100 cycles at 0.2 C, which is well comparable to the commercial Li-ion batteries. More surprisingly, an ultrahigh initial capacity of 8.41 mAh cm^–2^ is acquired under sulfur loading of 8.5 mg cm^–2^, corresponding to a low E/S of 4.1 μL mg^–1^ and N/P ratio of 2.1:1. And a desirable reversible capacity of 6.53 mAh cm^–2^ is ultimately retained after cycling (as summarized in Table S7) [57]. To further explore the feasibility of 3DIO FCSe-QDs@NC in practice, the pouch cell based on the 3DIO FCSe-QDs@NC hosts was fabricated. As exhibited in Fig. 7g, after pre-cycling at 0.05C for three cycles, the pouch cell delivers a high initial capacity of 985 mAh g^–1^ at 0.3C and shows excellent cycling stability as well as mitigatory polarization in charge/discharge curves. Moreover, the pouch cell can charge a mobile phone and also light a “Li–S” panel composed of 31 LED bulbs easily (Fig. S35 and Video S1).

Overall, these outstanding results collectively demonstrate the great benefits of the 3DIO FCSe-QDs@NC in promoting the high-efficiency synergy between the cathode and anode. Summarize from the aspect of material design, such multifunctional nanocomposites provide multiple advantages: (1) The highly dispersed FCSe-QDs with excellent sulfiphilic/lithiophilic properties can not only immobilize the LiPSs effectively but also regulate the Li deposition behaviors to stable Li anode. (2) The ordered interconnected conductive skeletons provide homogeneous electric field distribution and Li^+^ ion flow as well as smooth transportation pathways for electrons/ions to improve the redox kinetics. (3) Such hierarchical porous skeleton endows hosts with excellent capability for sulfur loading and exposure of active QDs sites for redox reactions and further mitigates the volume change of the sulfur and Li upon cycling.

## Conclusions

In summary, a 3DIO-ordered porous carbon matrix anchored with highly dispersed FCSe QDs was designed and employed as bi-served hosts for advanced sulfur cathode and dendrite-free Li anode. Experimental results together with theoretical calculations unveil that the abundant FCSe QDs possess a strong chemical affinity with LiPSs that can effectively capture soluble sulfur species, and the smooth diffusion-conversion of LiPSs is synchronously accelerated owing to the excellent catalytic activity. Additionally, the ordered 3DIO networks with sufficient void space and favorable lithiophilic features could regulate the Li nucleation/deposition behavior, thus suppressing the uncontrolled dendrites growth. As a consequence, the full cell constructed by the 3DIO FCSe-QDs@NC dual-functional host shows impressive electrochemical performances including the stable long cyclic ability (decay rate of 0.014% per cycle within 2,000 cycles at 2C), superior rate capability (2.61 mAh cm^–2^ at 5C with sulfur loading of 3.6 mg cm^–2^) and remarkable area capacity of 8.41 mAh cm^–2^ at 8.5 mg cm^–2^ with a low N/P of 2.1:1. Overall, this work provides a new perspective on the design of “two-in-one” host from systematic theoretical and experimental analysis, which can concurrently tackle the obstacles in both sulfur cathode and Li anode and propel the practical application of Li–S batteries.

### Supplementary Information

Below is the link to the electronic supplementary material.

Supplementary file1 (MP4 8910 kb)

Supplementary file2 (PDF 3541 kb)

## References

[1] Liu YT, Liu S, Li GR, Gao XP (2021). Strategy of enhancing the volumetric energy density for lithium-sulfur batteries. Adv. Mater..

[2] Ye Z, Jiang Y, Li L, Wu F, Chen R (2021). Rational design of mof-based materials for next-generation rechargeable batteries. Nano-Micro Lett..

[3] Huang Y, Lin L, Zhang C, Liu L, Li Y (2022). Recent advances and strategies toward polysulfides shuttle inhibition for high-performance Li-S batteries. Adv. Sci..

[4] Ng SF, Lau MYL, Ong WJ (2021). Lithium-sulfur battery cathode design: tailoring metal-based nanostructures for robust polysulfide adsorption and catalytic conversion. Adv. Mater..

[5] Fang R, Chen K, Yin L, Sun Z, Li F (2019). The regulating role of carbon nanotubes and graphene in lithium-ion and lithium-sulfur batteries. Adv. Mater..

[6] Wang J, Han WQ (2021). A review of heteroatom doped materials for advanced lithium–sulfur batteries. Adv. Funct. Mater..

[7] Zhou XJ, Tian J, Wu QP, Hu JL, Li CL (2020). N/O dual-doped hollow carbon microspheres constructed by holey nanosheet shells as large-grain cathode host for high loading Li-S batteries. Energy Storage Mater..

[8] Z. Li, Q. Zhang, L. Hencz, J. Liu, P. Kaghazchi et al., Multifunctional cation-vacancy-rich ZnCo_2_O_4_ polysulfide-blocking layer for ultrahigh-loading Li-S battery. Nano Energy **89**(9), 106331 (2021). 10.1016/j.nanoen.2021.106331

[9] Wang P, Xi B, Huang M, Chen W, Feng J (2021). Emerging catalysts to promote kinetics of lithium–sulfur batteries. Adv. Energy Mater..

[10] P. Wang, Z. Zhang, N. Song, X. An, J. Liu et al., WP nanocrystals on n,p dual-doped carbon nanosheets with deeply analyzed catalytic mechanisms for lithium–sulfur batteries. CCS Chemistry **5**(2), 397–411 (2023). 10.31635/ccschem.022.202202163

[11] Tian S, Zeng Q, Liu G, Huang J, Sun X (2022). Multi-Dimensional composite frame as bifunctional catalytic medium for ultra-fast charging lithium-sulfur battery. Nano-Micro Lett..

[12] Z. Gu, C. Cheng, T. Yan, G. Liu, J. Jiang et al., Synergistic effect of Co_3_Fe_7_ alloy and N-doped hollow carbon spheres with high activity and stability for high-performance lithium-sulfur batteries. Nano Energy **86**(7), 106111 (2021). 10.1016/j.nanoen.2021.106111

[13] Wang P, Xi B, Zhang Z, Huang M, Feng J (2021). Atomic tungsten on graphene with unique coordination enabling kinetically boosted lithium-sulfur batteries. Angew. Chem. Int. Ed..

[14] Sun W, Song Z, Feng Z, Huang Y, Xu ZJ (2022). Carbon-nitride-based materials for advanced lithium-sulfur batteries. Nano-Micro Lett..

[15] Ye Z, Jiang Y, Li L, Wu F, Chen R (2020). A high-efficiency CoSe electrocatalyst with hierarchical porous polyhedron nanoarchitecture for accelerating polysulfides conversion in li-s batteries. Adv. Mater..

[16] Tian Y, Li G, Zhang Y, Luo D, Wang X (2020). Low-bandgap Se-deficient antimony selenide as a multifunctional polysulfide barrier toward high-performance lithium-sulfur batteries. Adv. Mater..

[17] Yang D, Zhang C, Biendicho J, Han X, Liang Z (2020). ZnSe/N-doped carbon nanoreactor with multiple adsorption sites for stable lithium-sulfur batteries. ACS Nano.

[18] Meng L, Yao Y, Liu J, Wang Z, Qian D (2020). MoSe_2_ nanosheets as a functional host for lithium-sulfur batteries. J. Energy Chem..

[19] Yang D, Liang Z, Zhang C, Biendicho JJ, Botifoll M (2021). NbSe_2_ meets C_2_N: a 2D–2D heterostructure catalysts as multifunctional polysulfide mediator in ultra-long-life lithium–sulfur batteries. Adv. Energy Mater..

[20] Jiang YC, Arshad HMU, Li HJ, Liu S, Li GR (2021). Crystalline multi-metallic compounds as host materials in cathode for lithium-sulfur batteries. Small.

[21] Hu Y, Chen W, Lei T, Zhou B, Jiao Y (2019). Carbon quantum dots-modified interfacial interactions and ion conductivity for enhanced high current density performance in lithium-sulfur batteries. Adv. Energy Mater..

[22] Shen T, Yang LP, Pam ME, Shi YM, Yang HY (2020). Quantum dot-carbonaceous nanohybrid composites: preparation and application in electrochemical energy storage. J. Mater. Chem. A.

[23] Liu Y, Ma Z, Yang G, Wu Z, Li Y (2021). Multifunctional ZnCo_2_O_4_ quantum dots encapsulated in carbon carrier for anchoring/catalyzing polysulfides and self-repairing lithium metal anode in lithium-sulfur batteries. Adv. Funct. Mater..

[24] Xu ZL, Lin S, Onofrio N, Zhou L, Shi F (2018). Exceptional catalytic effects of black phosphorus quantum dots in shuttling-free lithium sulfur batteries. Nat. Commun..

[25] An Y, Luo C, Yao D, Wen S, Zheng P (2021). Natural cocoons enabling flexible and stable fabric lithium–sulfur full batteries. Nano-Micro Lett..

[26] Chen WJ, Li BQ, Zhao CX, Zhao M, Yuan TQ (2020). Electrolyte regulation towards stable lithium-metal anodes in lithium-sulfur batteries with sulfurized polyacrylonitrile cathodes. Angew. Chem. Int. Ed..

[27] Liu Y, Gao D, Xiang H, Feng X, Yu Y (2021). Research progress on copper-based current collector for lithium metal batteries. Energy Fuels.

[28] Park S, Jin HJ, Yun YS (2020). Advances in the design of 3D-structured electrode materials for lithium-metal anodes. Adv. Mater..

[29] X. Gao, B. Wang, Y. Zhang, H. Liu, H. Liu et al., Graphene-scroll-sheathed α-MnS coaxial nanocables embedded in N, S Co-doped graphene foam as 3D hierarchically ordered electrodes for enhanced lithium storage. Energy Storage Mater. **16**(8), 46–55 (2019). 10.1016/j.ensm.2018.04.027

[30] K. Liu, Z. Li, W. Xie, J. Li, D. Rao et al., Oxygen-rich carbon nanotube networks for enhanced lithium metal anode. Energy Storage Mater. **15**(5), 308–314 (2018). 10.1016/j.ensm.2018.05.025

[31] Duan J, Zheng Y, Luo W, Wu W, Wang T (2020). Is graphite lithiophobic or lithiophilic?. Natl. Sci. Rev..

[32] Yan X, Lin L, Chen Q, Xie Q, Qu B (2021). Multifunctional roles of carbon-based hosts for Li-metal anodes: A review. Carbon Energy.

[33] Ghimire P, Jaroniec M (2021). Renaissance of Stober method for synthesis of colloidal particles: New developments and opportunities. J. Colloid Interface Sci..

[34] Chen L, Xu Y, Cao G, Sari HMK, Duan R (2022). Bifunctional catalytic effect of cose_2_ for lithium-sulfur batteries: single doping versus dual doping. Adv. Funct. Mater..

[35] Adekoya D, Qian S, Gu X, Wen W, Li D (2020). DFT-guided design and fabrication of carbon-nitride-based materials for energy storage devices: a review. Nano-Micro Lett..

[36] Jiang B, Tian D, Qiu Y, Song X, Zhang Y (2021). High-index faceted nanocrystals as highly efficient bifunctional electrocatalysts for high-performance lithium-sulfur batteries. Nano-Micro Lett..

[37] Wang R, Wu R, Ding C, Chen Z, Xu H (2021). Porous carbon architecture assembled by cross-linked carbon leaves with implanted atomic cobalt for high-performance li-s batteries. Nano-Micro Lett..

[38] Liu D, Zhang C, Zhou G, Lv W, Ling G (2018). Catalytic effects in lithium-sulfur batteries: promoted sulfur transformation and reduced shuttle effect. Adv. Sci..

[39] Qiao Z, Zhang Y, Meng Z, Xie Q, Lin L (2021). Anchoring polysulfides and accelerating redox reaction enabled by fe-based compounds in lithium-sulfur batteries. Adv. Funct. Mater..

[40] Yuan Z, Peng HJ, Hou TZ, Huang JQ, Chen CM (2016). Powering lithium-sulfur battery performance by propelling polysulfide redox at sulfiphilic hosts. Nano Lett..

[41] Yang D, Li M, Zheng X, Han X, Zhang C (2022). Phase engineering of defective copper selenide toward robust lithium-sulfur batteries. ACS Nano.

[42] Wang P, Xu T, Xi B, Yuan J, Song N (2022). A Zn8 double-cavity metallacalix[8]arene as molecular sieve to realize self-cleaning intramolecular tandem transformation of Li-S chemistry. Adv. Mater..

[43] Wang H, Cui Z, He SA, Zhu J, Luo W (2022). Construction of ultrathin layered mxene-tin heterostructure enabling favorable catalytic ability for high-areal-capacity lithium-sulfur batteries. Nano-Micro Lett..

[44] Zhang ZW, Peng HJ, Zhao M, Huang JQ (2018). Heterogeneous/homogeneous mediators for high-energy-density lithium-sulfur batteries: progress and prospects. Adv. Funct. Mater..

[45] Fan FY, Carter WC, Chiang YM (2015). Mechanism and kinetics of Li_2_S precipitation in lithium-sulfur batteries. Adv. Mater..

[46] D.Q. Cai, J.L. Yang, T. Liu, S.X. Zhao, G.Z. Cao, Interfaces-dominated Li_2_S nucleation behavior enabled by heterostructure catalyst for fast kinetics Li-S batteries. Nano Energy **89**(25), 106452 (2021). 10.1016/j.nanoen.2021.106452

[47] Han Z, Zhao S, Xiao J, Zhong X, Sheng J (2021). Engineering d-p orbital hybridization in single-atom metal-embedded three-dimensional electrodes for li-s batteries. Adv. Mater..

[48] By.A. Bewic, M. Fleischm, H.R. Thirs, Kinetics of the electrocrystallization of thin films of calomel. Trans. Faraday Soc. 2200 (1962)

[49] Waluś S, Barchasz C, Bouchet R, Leprêtre J-C, Colin J-F (2015). Lithium/sulfur batteries upon cycling: structural modifications and species quantification by in situ and operando X-ray diffraction spectroscopy. Adv. Energy Mater..

[50] Ma C, Zhang Y, Feng Y, Wang N, Zhou L (2021). Engineering Fe-N coordination structures for fast redox conversion in lithium-sulfur batteries. Adv. Mater..

[51] Zhang C, Du R, Biendicho JJ, Yi M, Xiao K (2021). Tubular CoFeP@CN as a mott–schottky catalyst with multiple adsorption sites for robust lithium−sulfur batteries. Adv. Energy Mater..

[52] Lin L, Liu F, Zhang Y, Ke C, Zheng H (2022). Adjustable mixed conductive interphase for dendrite-free lithium metal batteries. ACS Nano.

[53] Jiang Z, Guo H, Zeng Z, Han Z, Hu W (2020). Reconfiguring organosulfur cathode by over-lithiation to enable ultrathick lithium metal anode toward practical lithium-sulfur batteries. ACS Nano.

[54] Zhang R, Chen XR, Chen X, Cheng XB, Zhang XQ (2017). Lithiophilic sites in doped graphene guide uniform lithium nucleation for dendrite-free lithium metal anodes. Angew. Chem. Int. Ed..

[55] A. Pei, G. Zheng, F. Shi, Y. Li, Y. Cui Nanoscale nucleation and growth of electrodeposited lithium metal. Nano Lett. **17**(2), 1132–1139 (2017). 10.1021/acs.nanolett.6b04755 10.1021/acs.nanolett.6b04755 28072543

[56] G. Zhou, H. Chen, Y. Cui Formulating energy density for designing practical lithium–sulfur batteries. Nat. Energy **7**(4), 312–319 (2022). 10.1038/s41560-022-01001-0

[57] Huang Y, Hu X, Li J, Zhang J, Cai D (2020). Rational construction of heterostructured core-shell Bi_2_S_3_@Co_9_S_8_ complex hollow particles toward high-performance Li- and Na-ion storage. Energy Storage Mater..

