# Supplementary material for: Dual-Functional Lithiophilic/Sulfiphilic Binary-Metal Selenide Quantum Dots Toward High-Performance Li–S Full Batteries
Source: Nanomicro Lett. 2023 Mar 15;15:67. doi: 10.1007/s40820-023-01037-1 (PMC10014643; doi:10.1007/s40820-023-01037-1)
Supplement: Supplementary file 2 — Supplementary file2 (PDF 3541 kb) [file 40820_2023_1037_MOESM2_ESM.pdf]

Supporting Information for

# Dual-Functional Lithiophilic/Sulfiphilic Binary-Metal Selenide Quantum Dots toward High-Performance Li–S Full Batteries

Youzhang Huang[1], Liang Lin[1], Yinggan Zhang[1], Lie Liu[1], Baisheng Sa[3], Jie Lin[1], Laisen Wang [1,*], Dong-Liang Peng[1,*], Qingshui Xie[1,2,*]

[1] State Key Lab for Physical Chemistry of Solid Surfaces, Fujian Key Laboratory of Surface and Interface Engineering for High Performance Materials, College of Materials, Xiamen University, Xiamen 361005, P. R. China

[2] Shenzhen Research Institute of Xiamen University, Shenzhen, 518000, P. R. China

[3] Multiscale Computational Materials Facility, College of Materials Science and Engineering, Fuzhou University, Fuzhou 350100, P. R. China

*Corresponding authors. E-mail: wangls@xmu.edu.cn (L. Wang), xieqsh@xmu.edu.cn (Q. Xie), dlpeng@xmu.edu.cn (D.-L. Peng)

## S1 Experimental Section

### S1.1 Characterization

X-ray diffraction (XRD) patterns of acquired samples were measured by Bruker AXS D8 Advance X-ray diffractometer with Cu $K_\alpha$ radiation operating at 40 kV and 40 mA. The morphology and microstructures were examed by TEM (TECNAI F-30) operated at 300 kV and SEM (Hitachi SU-70) equipped with an energy-dispersive X-ray spectroscopy (EDS) detector operated at 5 kV. The content of sulfur in the composites was measured by TGA experiment (SDT-Q600 thermal analyzer) at a heating rate of 10 °C min$^{-1}$ in an $N_2$ atmosphere. XPS analysis was carried
out on PHI QUANTUM 2000 (monochromatic Al K X-ray source). Raman spectra were performed using an Xplora Raman microscope with an excitation wavelength of 785 nm. UV-vis absorption tests were performed with UV-vis-near-infrared spectrophotometry (UV-Vis, Lambda 750, PerkinElmer). The specific surface area and analysis of the pore size distribution were obtained from nitrogen adsorption-desorption isotherms (BSD-3H-2000PM2).

### S1.2 Adsorption and Catalytic Studies

Sulfur and $Li_2S$ (99.9%, Alfa Aesar) were dissolved in appropriate amounts of DME/DOL (volume ratio of 1:1) solution with a molar ratio of 5:1, and then the mixture solution was vigorously magnetic stirred overnight in an Ar-filled glovebox until a dark brown solution was obtained. 20 mg of the adsorbent (3DIO FCSe-QDs@NC, FCSe-QDs@NC, and 3DIO NC cathodes) was poured into a 3.0 mL of 10 mM $Li_2S_6$ solution to observe the color change of the mixture solution.

For the symmetric cell assembly and measurements, the working electrode was prepared by mixing host matrices materials and PVDF at a weight ratio of 9:1. Symmetric cells were assembled by using two identical electrodes with a Celgard 2400 membrane as the separator, and 60 μL of $Li_2S_6$ electrolyte (containing 1 mol $L^{-1}$ LiTFSI, 2 wt% $LiNO_3$, and 0.2 M $Li_2S_6$ in DOL/DME solution with a volume ratio of 1:1) as electrolyte. The CV curves of symmetric cells were performed within the voltage range of -1.0–1.0 V (*vs.* $Li^+$/Li). EIS was tested by Autolab electrochemical workstation (NOVA 1.9) with a frequency ranging from 0.01 Hz to 100 kHz.

For the $Li_2S$ nucleation and decomposition measurement, the cells were assembled by the above active electrodes as working electrodes and Li foils as counter electrodes. 25 μL of 0.5 M $Li_2S_8$ and 1.0 M LiTFSI dissolved in a tetraglyme solution were used as the catholyte, while 25 μL of 1.0 M LiTFSI dissolved in a tetraglyme solution as the anolyte. For the nucleation test, the cells were galvanostatically discharged to 2.06 V at 0.112 mA and then kept potentiostatically at 2.05 V until the current dropped below $10^{-5}$ A. For the decomposition process, the cells were galvanostatically discharged to 1.7 V at a constant current of 0.10 mA, then continue galvanostatically discharged to 1.7 V at 0.01 mA, and final potentiostatically charged at 2.40 V for 20,000 s.

For the LSV measurements, a three-electrode configuration was fabricated using glassy carbon coated with active material as the working electrode, Ag/AgCl electrode as the reference electrode, platinum sheet as the counter electrode, and 0.1 mol $L^{-1}$ $Li_2S$/methanol solution as the electrolyte. The LSV tests were conducted using the CHI660D electrochemical workstation (Shanghai Chenhua Device Company, China) from -0.8 V to -0.1V at a scan rate of 5 mV $s^{-1}$.

### S1.3 Electrochemical Measurements

For the sulfur cathode test, the resulting cathode electrodes, Li anode, and Celgard 2400 separator were employed to assemble the CR2032 coin-type Li-S batteries in an argon-filled glove box (<1 ppm of $O_2$ and $H_2O$). The electrolyte was lithium bis(trifluoromethanesulfonyl)imide (LiTFSI) (1 M) in a mixed solvent of 1,2-dimethoxyethane and 1,3-dioxolane (1:1, v/v) with 2 wt% of $LiNO_3$ additive, and the E/S ratio of half-cell tests was maintained at about 20 μL $mg^{-1}$. Cycling and rate performance tests in the cutoff potential of 1.8–2.7 V (vs $Li^+$/Li) were performed on a Neware battery test system (CT-4008, 5 V-10 mA, and 5 V-50 mA). CV curves were recorded in the voltage range from 1.7–2.8 V (vs $Li^+$/Li).

For the synthesis of Li metal hybrid anodes, the 3DIO FCSe-QDs@NC (or FCSe-QDs@NC and 3DIO NC) was firstly mixed with PVDF in NMP solvent with a mass ratio of 9:1, coated onto the Cu foils, and cut into discs with the mass loading of about 1.5 mg $cm^{-2}$. The Li/3DIO FCSe-QDs@NC, Li/FCSe-QDs@NC, Li/3DIO NC, and Li@Cu anodes were prepared by electrochemical deposition. The deposition process was performed using CR2032 coin cells with Li foils as the counter electrode and the aforementioned Li–S battery electrolyte as the electrolyte. Prior to the test, the cell was cycled at 50 μA between 0.01 and 3.0 V (vs $Li^+$/Li) for 5 cycles to form a stable SEI film. For Li symmetric battery configurations, both working and reference electrodes are the Li-deposited electrodes. Then, 15 mAh $cm^{-2}$ of Li was deposited on the host through galvanostatic discharging. The cycling performances were recorded under a variety of current rates and capacities. The full cells were constructed by the obtained sulfur cathodes and the corresponding pre-deposited Li anodes with a N/P ratio of lower than 5 for routine tests (a Li deposition capacity of 12 mAh $cm^{-2}$).

## S1.4 Computational Simulation

The density functional theory (DFT) calculations were operated in the Vienna Ab-initio Simulation Package (VASP) [S1, S2]. The projector augmented waves (PAW) in the Perdew-Burke-Ernzerhof (PBE) form were chosen as the pseudopotentials [S3]. The Brillouin zone of the supercell was sampled by a 2 × 2 × 1 uniform k-point mesh. The energy cutoff of the plane base sets was 500 eV. All the atoms in the structures were relaxed until the residual forces were less than 0.01 eV $Å^{-1}$ and the total energy difference was less than $10^{-5}$ eV. The binding energy ($E_b$) was defined as the energy difference of adsorbed model ($E_{sur/Li_2S_n}$) and the summation of pure $Li_2S_n$ ($E_{Li_2S_n}$, $n$ = 1, 2, 4, 6, 8) molecule and the surface energy ($E_{sur}$) according to $E_b = E_{sur/Li_2S_n} - E_{Li_2S_n} - E_{sur}$, where more negative values indicated stronger binding interaction. The transition state of $Li_2S$ decomposition on the surface was located by the nudged elastic band (NEB) method. All the calculation models adopted in this work were conducted with the ALKEMIE platform [S4]. Charge density difference was obtained from the charge difference between the substrate and the adsorbent.

Theoretical expressions of the current-time transients of four classic electrochemical deposition models were presented as follows:

2DI: $$\frac{i}{i_m} = \left(\frac{t}{t_m}\right)\left\{exp\left[\frac{t_m^2-t^2}{2t_m^2}\right]\right\} \quad (S1)$$

2DP: $$\frac{i}{i_m} = \left(\frac{t}{t_m}\right)^2\left\{exp\left[\frac{-2(t^2-t_m^3)}{3t_m^2}\right]\right\} \quad (S2)$$

3DI: $$\frac{i}{i_m} = \left(\frac{1.9542}{t/t_m}\right)^{1/2}\left\{1-exp\left[-1.2564\left(\frac{t}{t_m}\right)\right]\right\} \quad (S3)$$

3DP: $$\frac{i}{i_m} = \left(\frac{1.2254}{t/t_m}\right)^{1/2}\left\{1-exp\left[-2.3367\left(\frac{t}{t_m}\right)^2\right]\right\} \quad (S4)$$

# Supplementary Figures and Tables

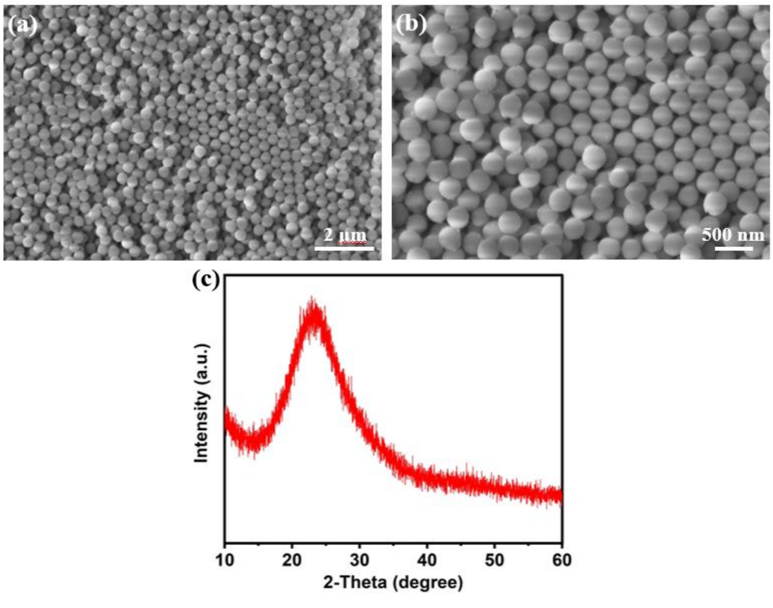


**Fig. S1** (**a, b**) SEM images and (**c**) XRD pattern of $SiO_2$ spheres

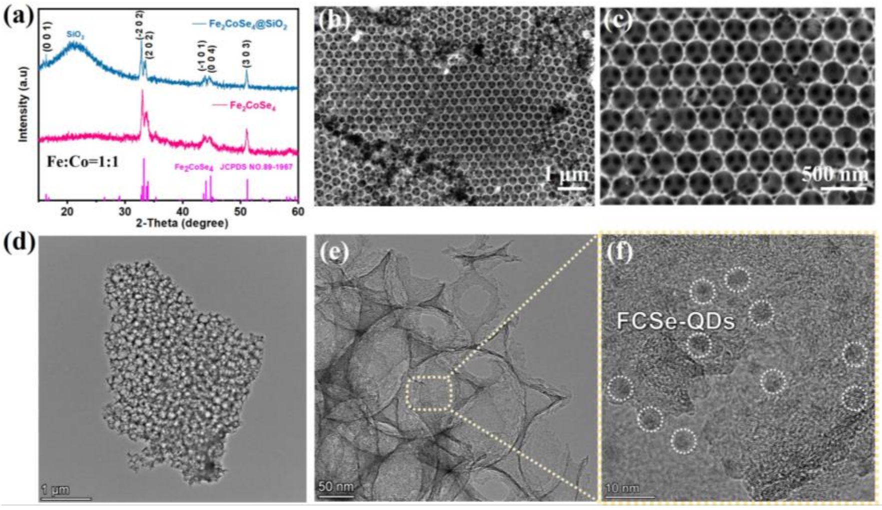


**Fig. S2** (**a**) XRD patterns of FCSe-QDs@NC@SiO$_2$ and 3DIO FCSe-QDs@NC. (**b,c**) SEM images and (**d-f**) TEM images of the 3DIO FCSe-QDs@NC

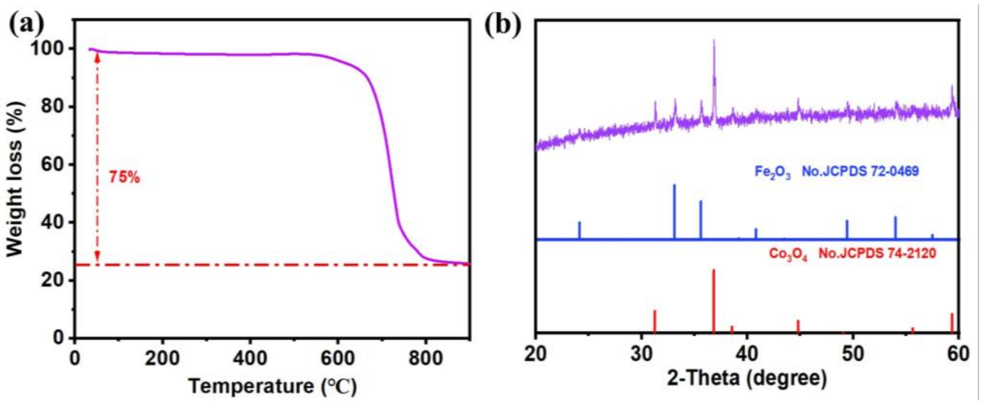


**Fig. S3** (**a**) TG curve of 3DIO FCSe-QDs@NC in the air with a heating rate of 10 °C min$^{-1}$. (**b**) XRD pattern of 3DIO FCSe-QDs@NC after calcination in air at 900 °C

The reaction can be written as:

$$6Fe_2CoSe_4 + 37O_2 \rightarrow 6Fe_2O_3 + 2Co_3O_4 + 24SeO_2$$

Therefore, the weight ratio of $Fe_2CoSe_4$ in 3DIO FCSe-QDs@NC is calculated to be about 81 wt%

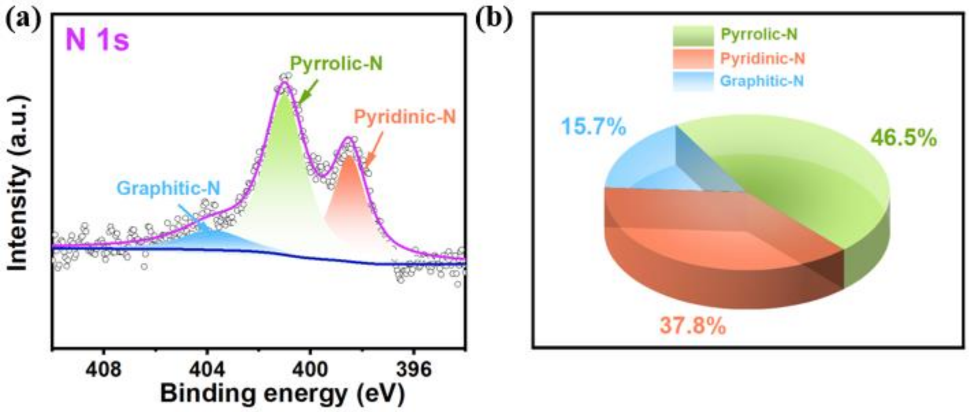


**Fig. S4** (**a**) High-resolution XPS spectra of N 1s. (**b**) The ratio of Pyrrolic-N, Pyridinic-N and Graphitic-N

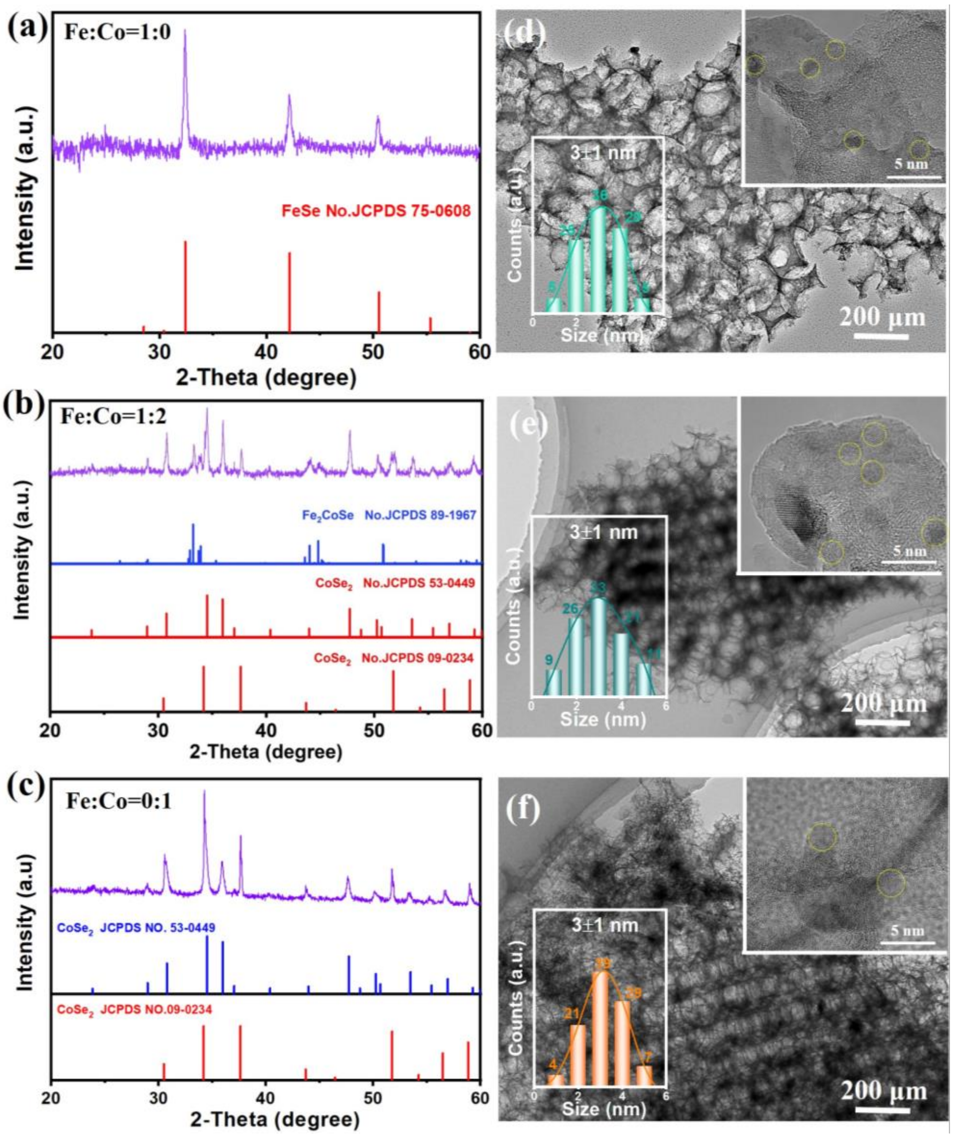


**Fig. S5** (**a-c**) XRD patterns and (**d-f**) TEM images of the final products obtained by varying the ratios of cobalt and iron, and the insets show the size distribution of QDs

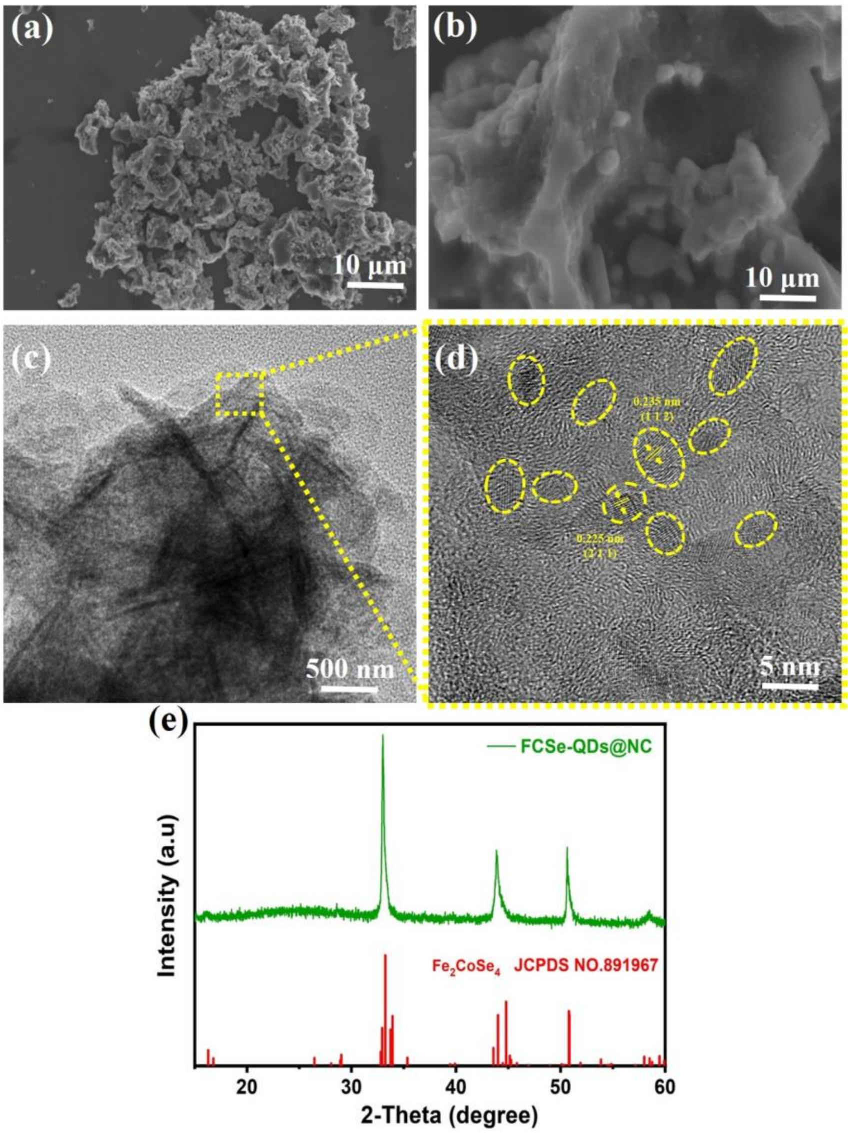


**Fig. S6** (**a, b**) SEM images, (**c, d**) TEM images, and (**e**) XRD pattern of FCSe-QDs@NC

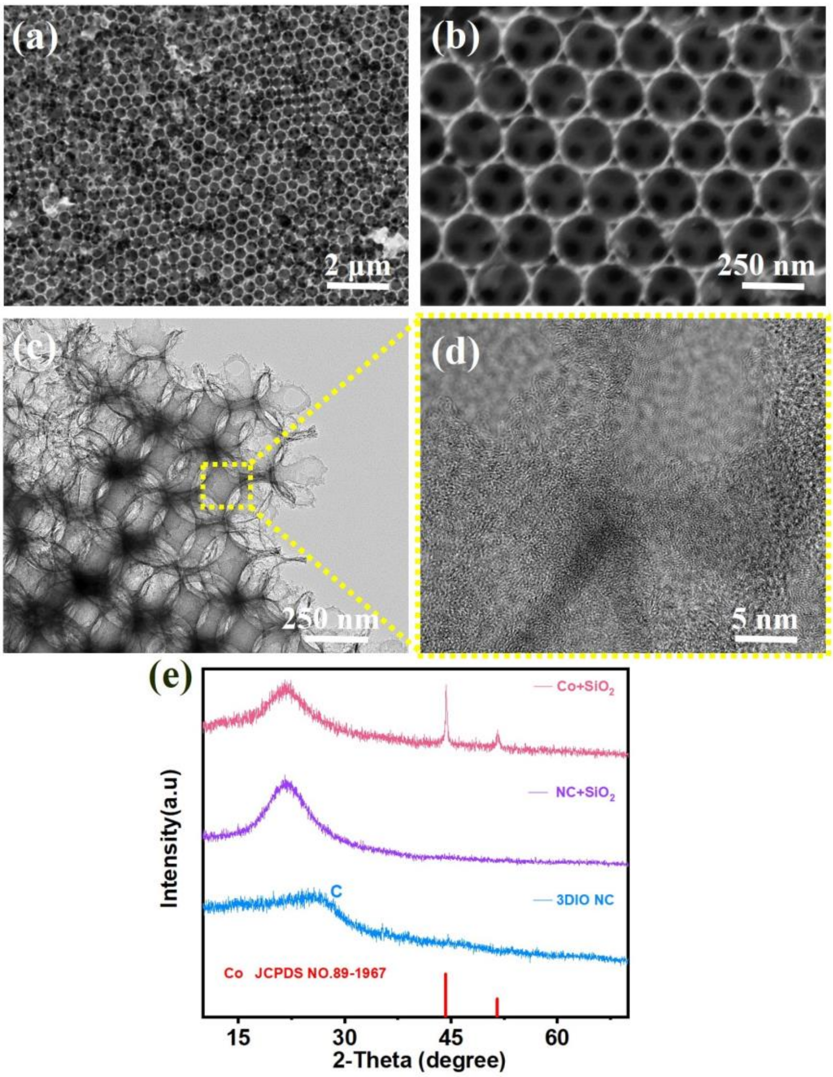


**Fig. S7** (**a, b**) SEM images, (**c, d**) TEM images, and (**e**) XRD patterns of Co-based oleate and $SiO_2$ mixture after calcination (Co+$SiO_2$), sample after acid treatment (NC+$SiO_2$), and 3DIO NC final product

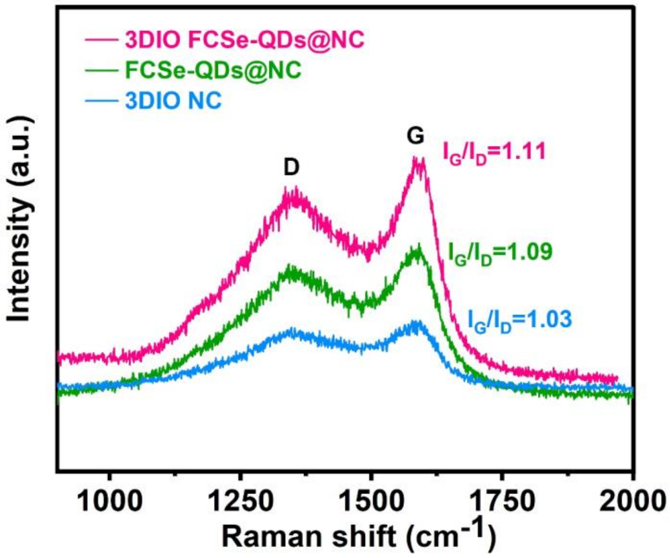


**Fig. S8** Raman spectra of 3DIO FCSe-QDs@NC, FCSe-QDs@NC, and 3DIO NC

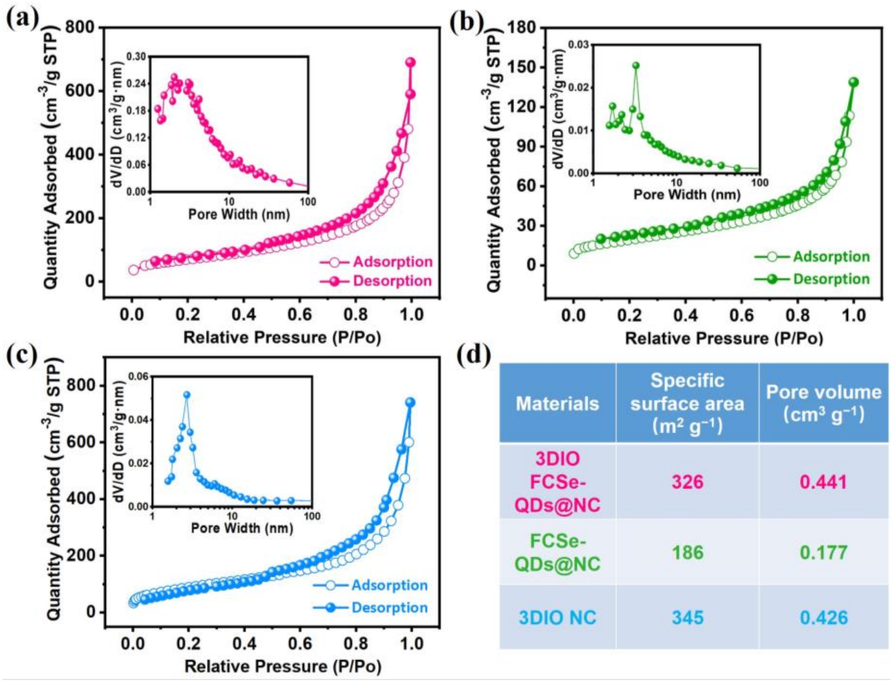


| Materials | Specific surface area ($m^2$ $g^{-1}$) | Pore volume ($cm^3$ $g^{-1}$) |
|---|---|---|
| 3DIO FCSe-QDs@NC | 326 | 0.441 |
| FCSe-QDs@NC | 186 | 0.177 |
| 3DIO NC | 345 | 0.426 |

**Fig. S9** (**a-c**) Nitrogen adsorption and desorption isotherms and (**d**) Supertable of 3DIO FCSe-QDs@NC, FCSe-QDs@NC and 3DIO NC

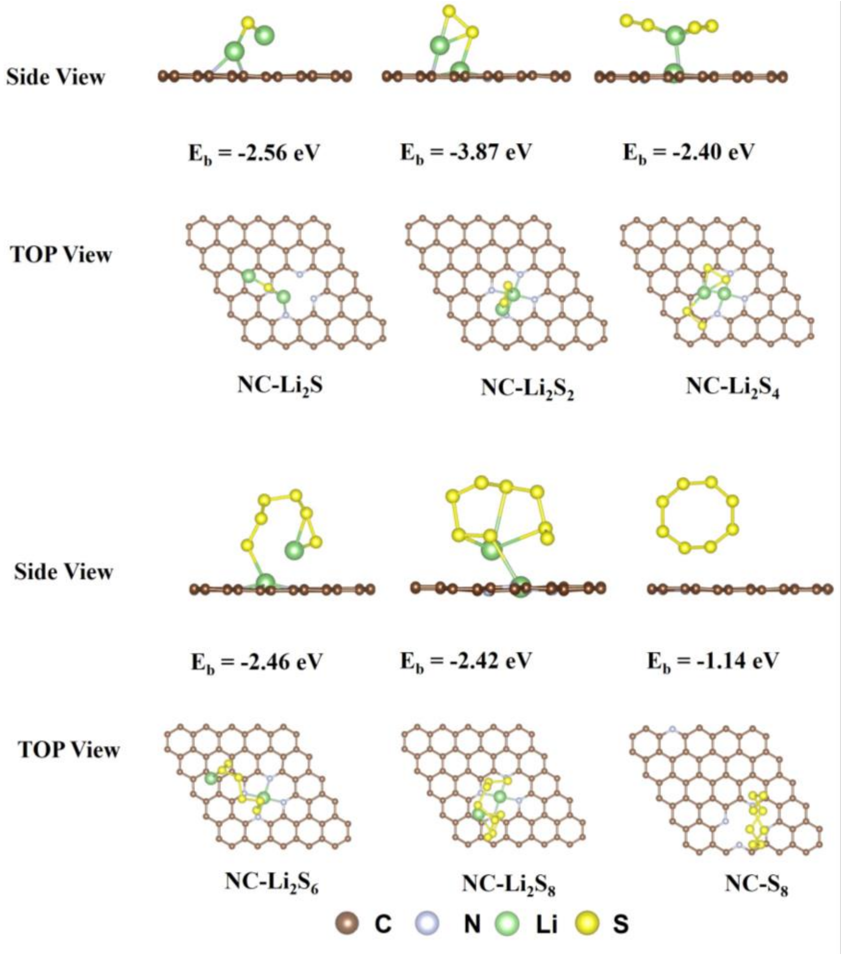


**Fig. S10** Binding energies and adsorbed structures of LiPSs on 3DIO NC based on DFT calculation

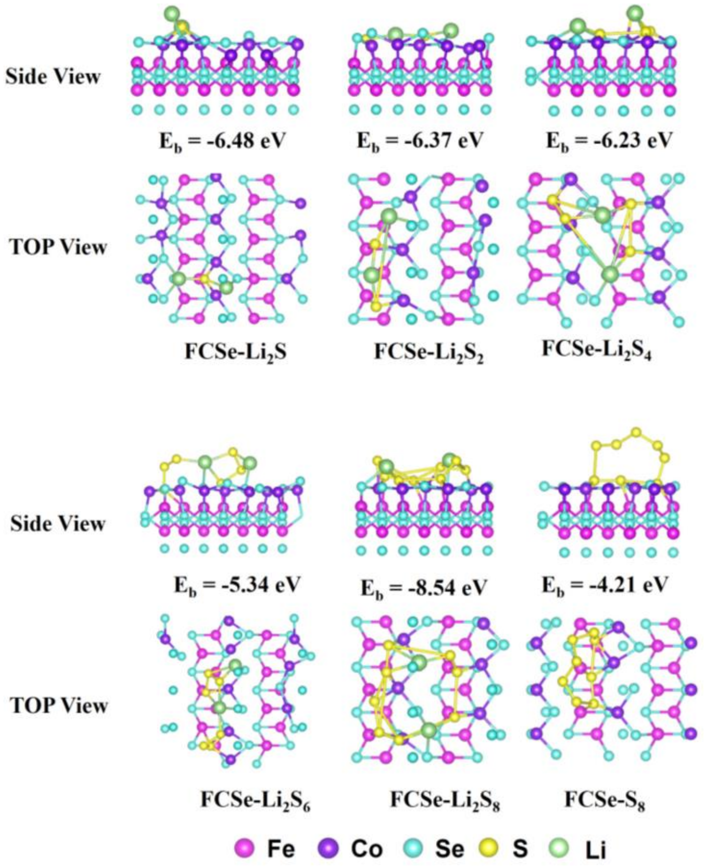


**Fig. S11** Binding energies and adsorbed structures of LiPSs on the (001) lattice plane of 3DIO FCSe-QDs@NC based on DFT calculations

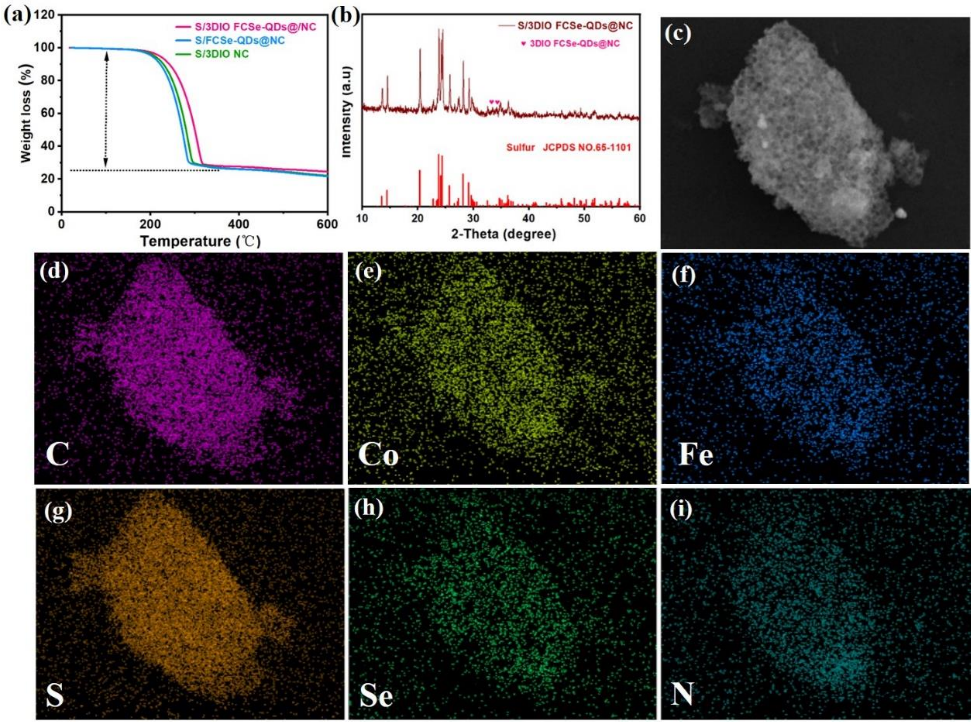


**Fig. S12** (**a**) TGA curves of S/3DIO FCSe-QDs@NC, S/FCSe-QDs@NC, and S/3DIO NC. (**b**) XRD patterns, (**c**) SEM image, and (**d-i**) corresponding EDS elemental mappings of S/3DIO FCSe-QDs@NC

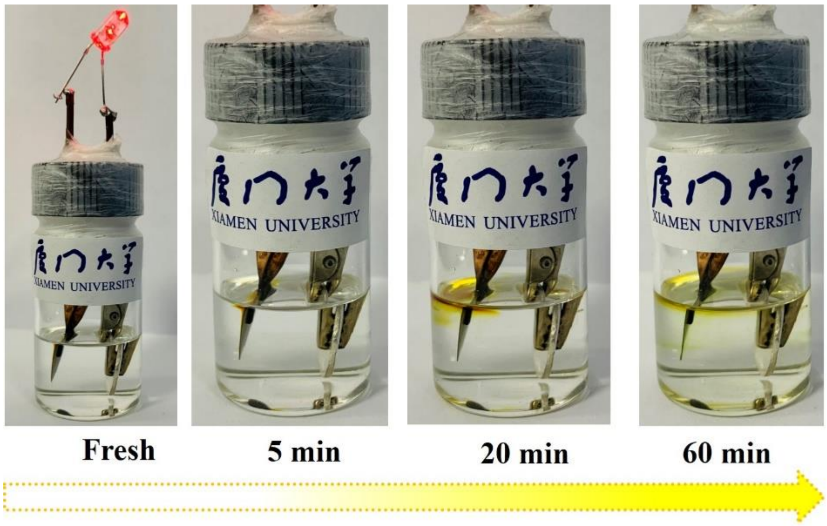


**Fig. S13** Visual illustration of polysulfide entrapment at different discharge stages for S/3DIO NC

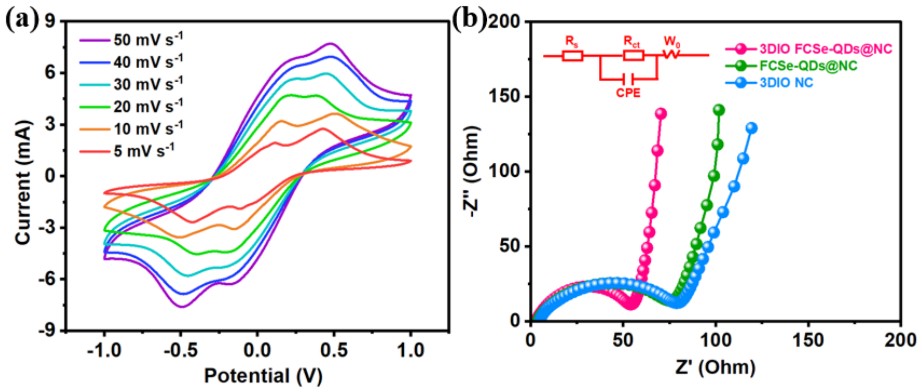


**Fig. S14** (**a**) CV curves of the 3DIO FCSe-QDs@NC-based symmetric cells at different scanning rates. (**b**) EIS spectra of three different symmetric cells. The inset is the corresponding equivalent circuit

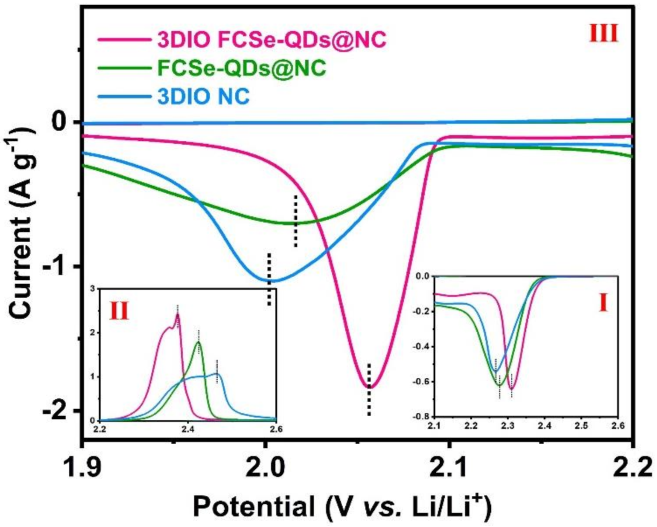


**Fig. S15** Local enlargement of different peaks in the CV curve from coin-type cells assembled with different sulfur cathodes

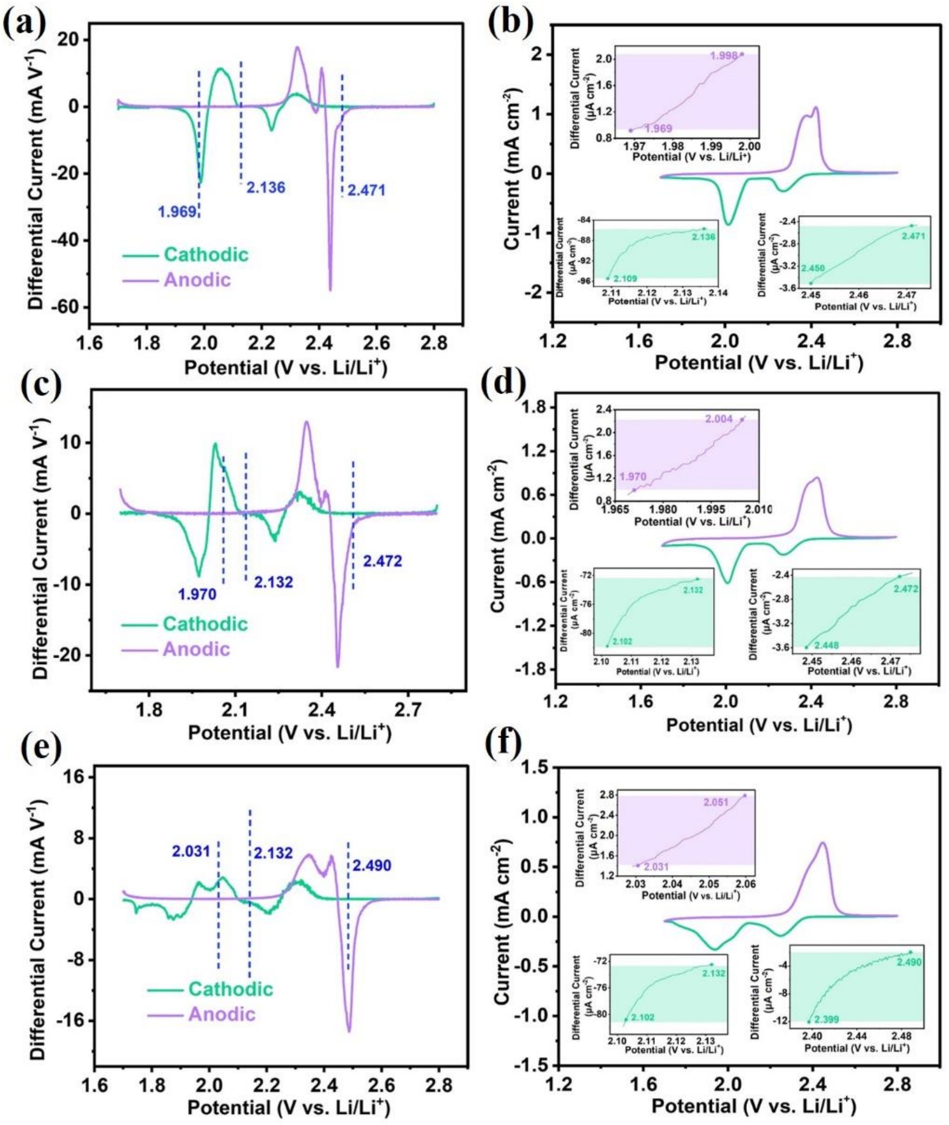

**Fig. S16** Onset potential tests for Li–S redox reactions. Differential CV curves with (**a, b**) 3DIO FCSe-QDs@NC, (**c, d**) FCSe-QDs@NC and (**e, f**) 3DIO NC

**Note:** The baseline voltage and current density are defined as the value before the redox peak, where the variation in current density is the smallest, namely dI/dV = 0. Baseline voltages are denoted in Cambridge blue for cathodic peak I, II, and in black for anodic peak III, respectively. The CV curves and corresponding onset current density is 10 μA $cm^{-2}$ beyond the corresponding baseline current density (more specifically, 10 μA $cm^{-2}$ more negative than baseline current density for the cathodic peaks or 10 μA $cm^{-2}$ more positive than baseline current density for anodic peaks). As shown in the inset, the baseline voltages are exhibited, and the colored region indicates the gap in current density (10 μA $cm^{-2}$) [S5].

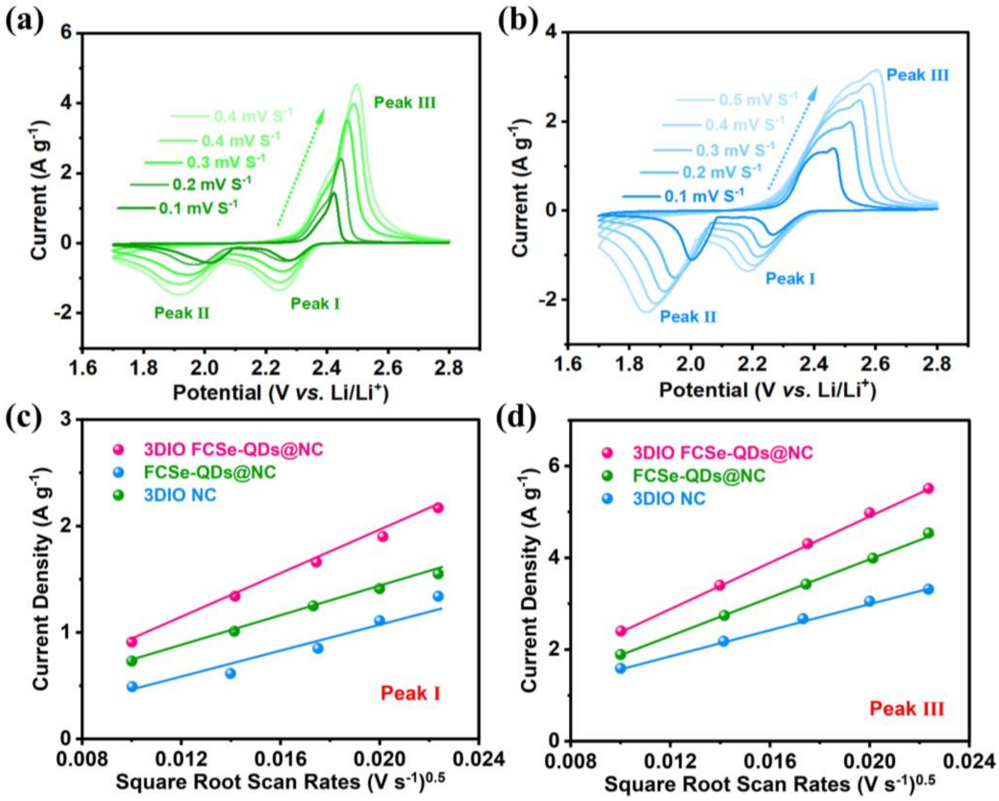


**Fig. S17** (**a, b**) CV curves at different scanning rates and (**c,d**) the linear fits of the peak currents *vs.* square root of scan rates from CV curves

Randles-Sevcik Equation [S6]:

$$I_p = (2.69 \times 10^5) n^{1.5} A D^{0.5} C v^{0.5}$$

Wherein $I_p$ is the peak current density, $n$ is the number of electrons during reactions, $A$ is the electrode area, $D_{Li^+}$ is the $Li^+$ ion diffusion coefficient, $C$ is the concentration of $Li^+$ ion in the electrolyte, and $v$ is the scanning rate. The higher the slope, the stronger the ion diffusion ability [S7, S8].

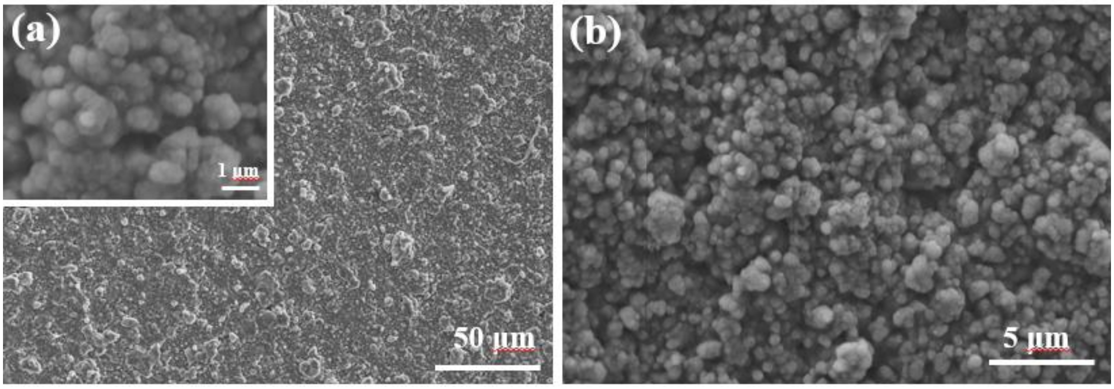


**Fig. S18** SEM images of the final $Li_2S$ deposited on 3DIO FCSe-QDs@NC electrode

**Note:** On the 3DIO FCSe-QDs@NC electrode surface, the $Li_2S$ exhibits 3D granular-morphology and is evenly deposited without obvious aggregation of large particles, which is mainly driven by the abundant catalytic sites in the 3D porous carbon skeleton. Moreover, the smooth LiPSs diffusion and efficient charge transfer profited from the conductive carbon skeleton contribute to the rapid $Li_2S$ nucleation and uniform

deposition on the $Li_2S$/host/electrolyte three-phase interface [S9]. In stark contrast, there are many sheet-like self-assembled $Li_2S$ agglomerates deposited on the bulk FCSe-QDs@NC electrode surface, on account of the limited amount of active sites exposed on bulk surfaces and rapidly depleted during the deposition process, which has been proved to be universal in ether-based electrolytes (Fig. S17) [S10]. For the catalyst-free, the deposited $Li_2S$ completely exhibits a 2D sheet-like morphology with a rough and uneven surface. And such sheet-like structures would lessen the three-phase interface mediated for the electron/ion transfer and further impedes the subsequent growth of $Li_2S$ (Fig. S18)

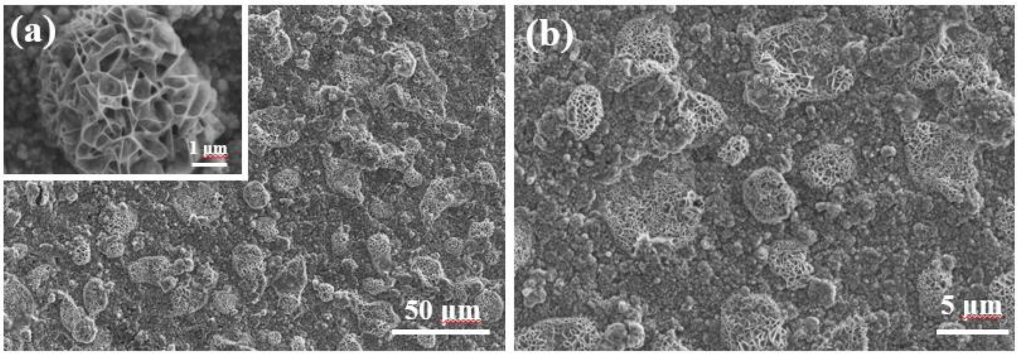


**Fig. S19** SEM images of the final $Li_2S$ deposited on FCSe-QDs@NC electrode

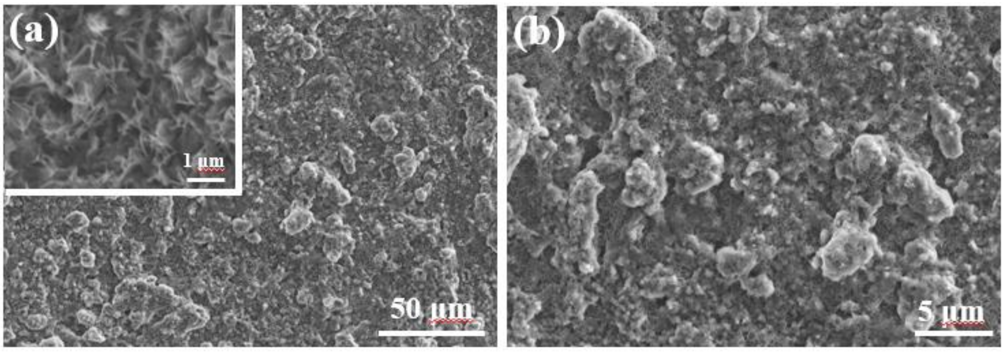


**Fig. S20** SEM images of the final $Li_2S$ deposited on the 3DIO NC electrode

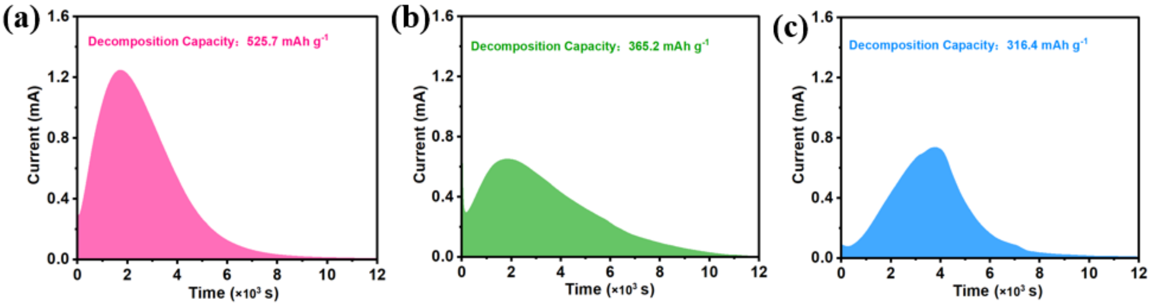


**Fig. S21** Potentiostatic charge profiles at 2.40 V for evaluating dissolution kinetics of $Li_2S$ for (**a**) 3DIO FCSe-QDs@NC, (**b**) FCSe-QDs@NC, and (**c**) 3DIO NC

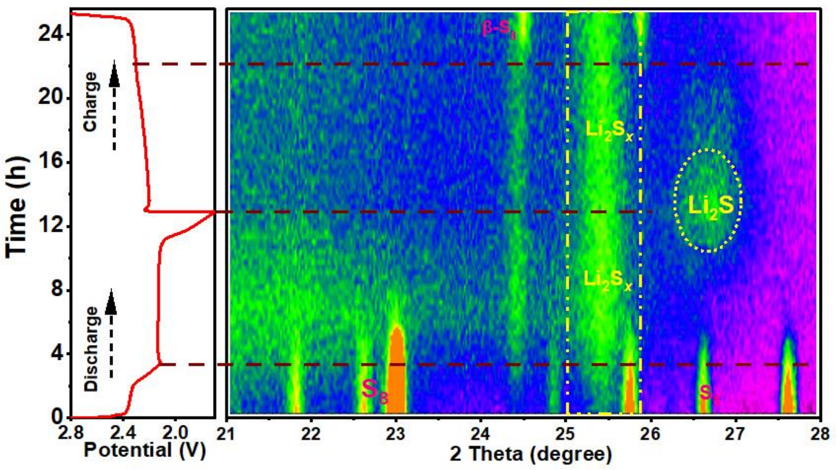


**Fig. S22** *In-situ* XRD pattern of 3DIO NC electrode

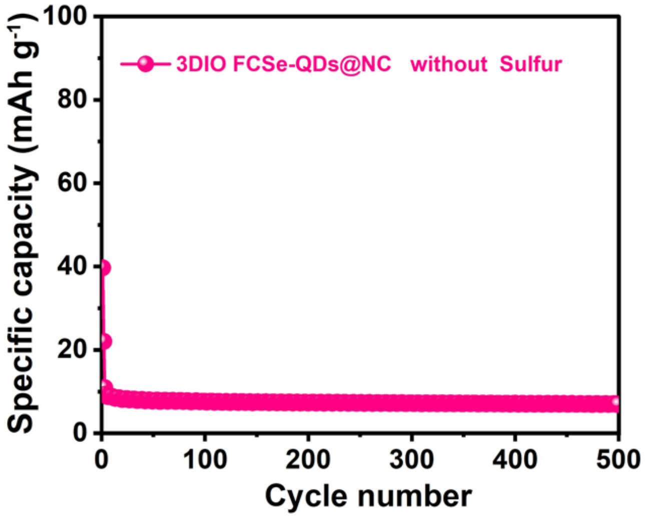


**Fig. S23** Cycling performance of the pure 3DIO FCSe-QDs@NC without sulfur loading at 0.2 C

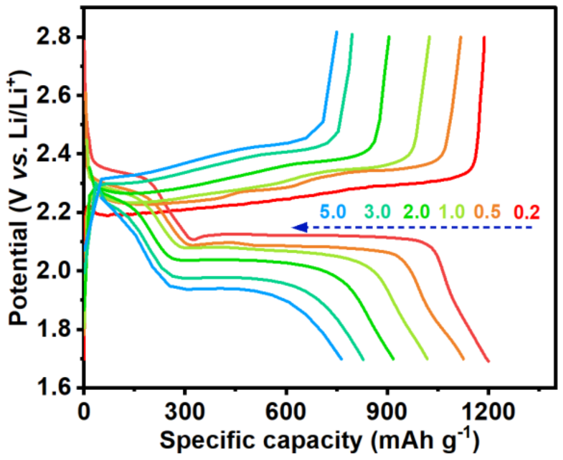


**Fig. S24** The discharge/charge curves of the 3DIO FCSe-QDs@NC cell at different current densities

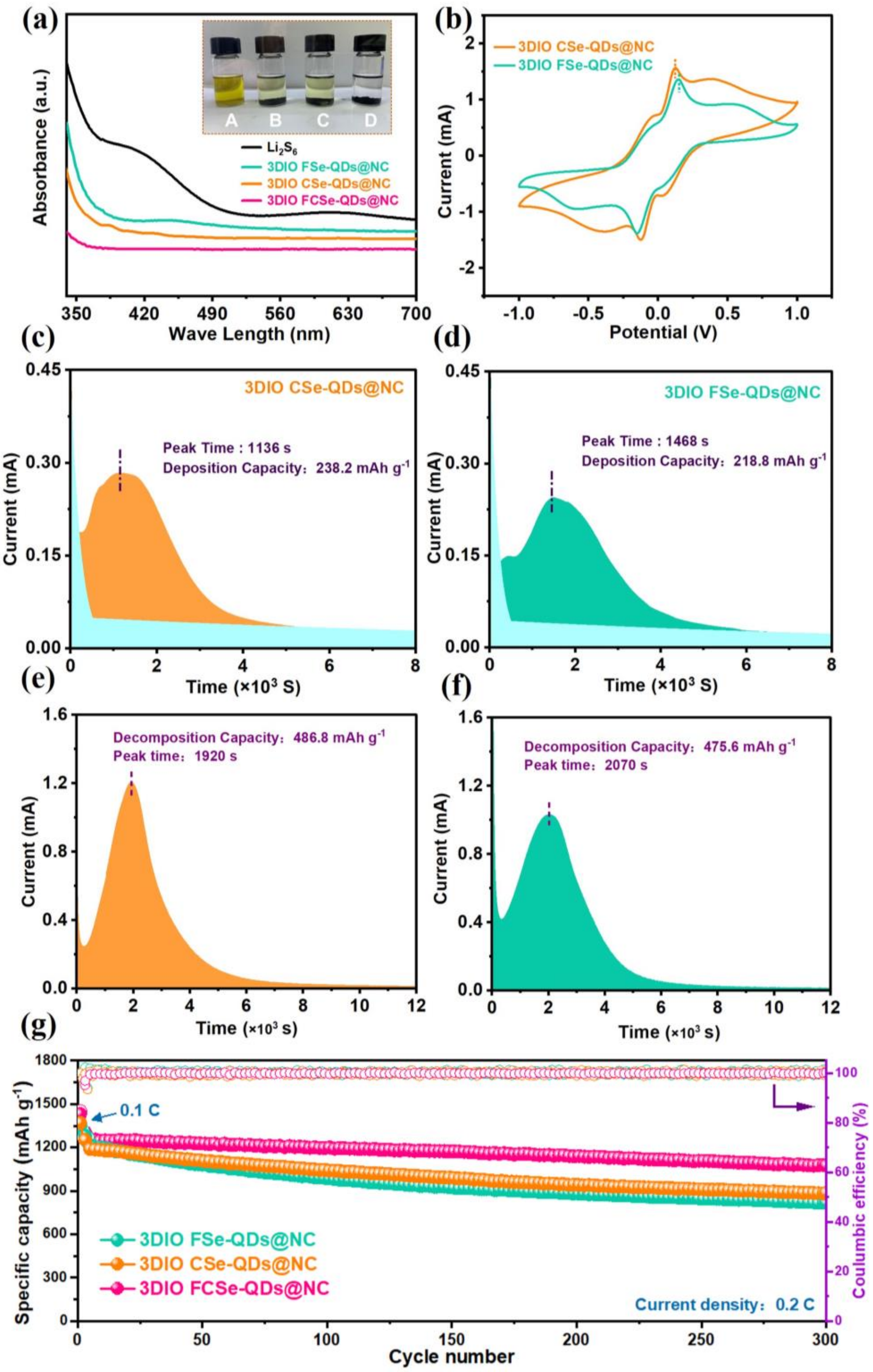


**Fig. S25** (**a**) Optical photograph and UV–vis spectra of $Li_2S_6$ solutions containing different adsorbents after resting for 6 h (A: Li2S6; B: 3DIO CSe-QDs@NC; C: 3DIO FSe-QDs@NC; D: 3DIO FCSe-QDs@NC). (**b**) CV curves of symmetric cells. (**c, d**) $Li_2S$ nucleation tests and (**e, f**) potentiostatic charge profiles. (**g**) Cycling performances and the corresponding coulombic efficiencies of Li–S batteries with S/3DIO FCSe-QDs@NC, S/CSe-QDs@NC, and S/3DIO S/FSe-QDs@NC electrodes at 0.2 C

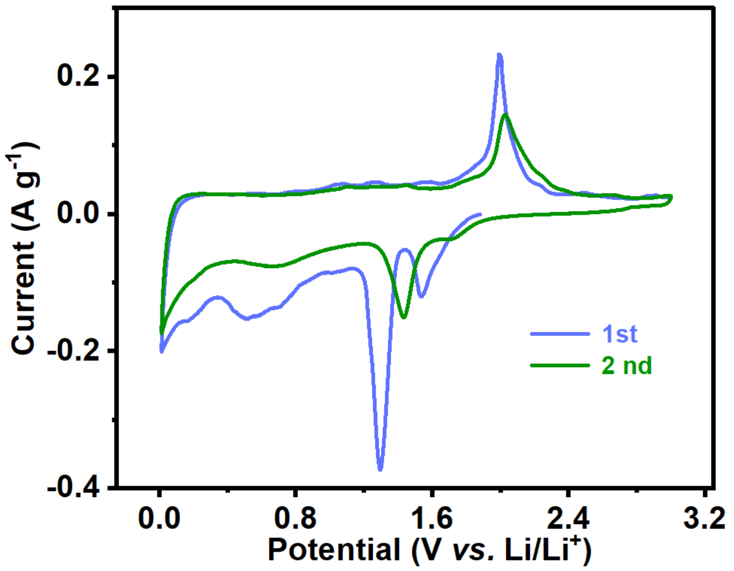


**Fig. S26** The cyclic voltammetry profiles of 3DIO FCSe-QDs@NC electrode at a scanning rate of 0.1 mV $s^{-1}$ between 0.01 and 3.0 V

**Note:** During the first cathodic scanning, three reduction peaks at 1.55, 1.29, and 0.51 V are ascribed to the initial insertion of $Li^+$ into the 3DIO FCSe-QDs@NC electrode, and finally result in the formation of Co, Fe, and $Li_2Se$, corresponding to the following reaction [S11, S12]:

$$Fe_2CoSe_4 + 8Li^+ \rightarrow 2Fe + Co + 4Li_2Se$$

The *in-situ* formed Co and Fe phase works as the preferred nucleation sites for the subsequent Li deposition. While the formed $Li_2Se$ phase possesses high ionic conductivity, which is favorable for fast Li-ion diffusion. Such mixed conductive phases have been proven to effectively regulate the nucleation and deposition of Li metal [S13-S15]

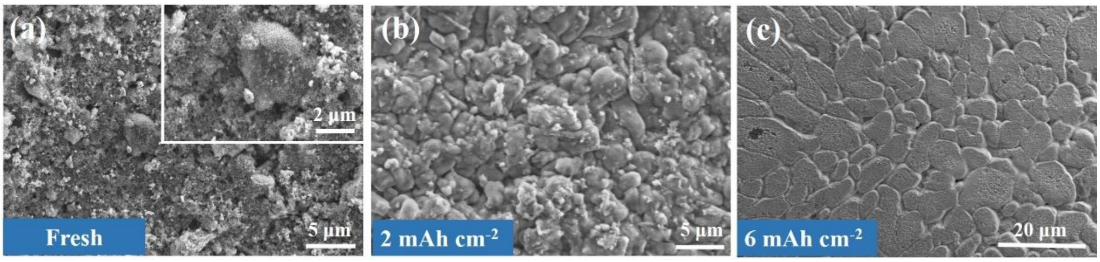


**Fig. S27** Morphological evolution of 3DIO FCSe-QDs@NC electrode at different states

**Note:** With the increase of Li deposition capacity, some smooth-surfaced moss-like Li deposits are tightly packed on the 3DIO FCSe-QDs@NC matrix, and no obvious Li dendrites are observed. This indicates that the 3D-ordered carbon framework combined with abundant lithophilic sites is beneficial to tuning the Li plating/stripping behavior to harvest dendrite-free Li anode.

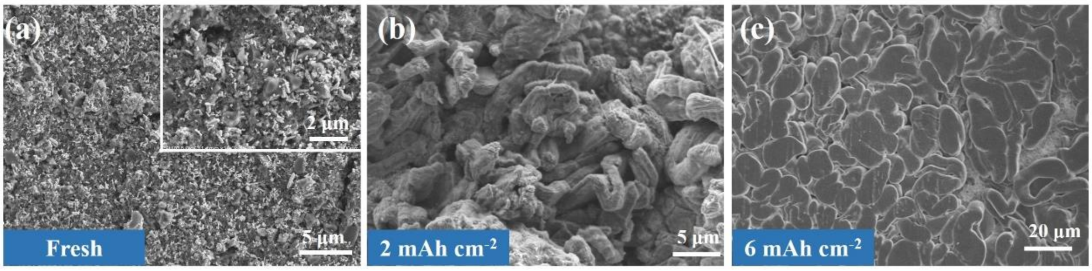


**Fig. S28** Morphological evolution of FCSe-QDs@NC electrode at different states

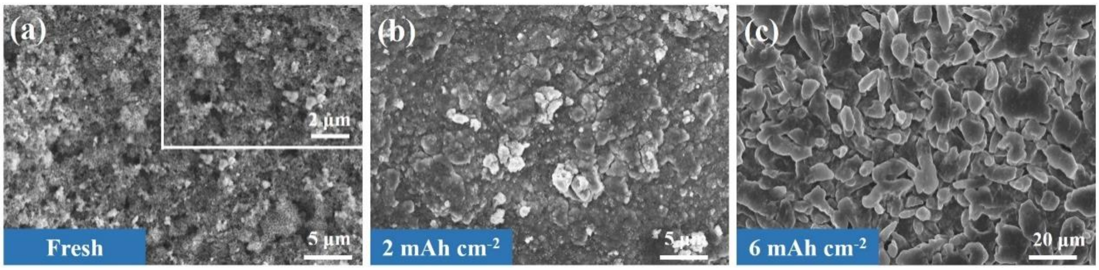


**Fig. S29** Morphological evolution of 3DIO NC electrode at different deposition capacities of Li

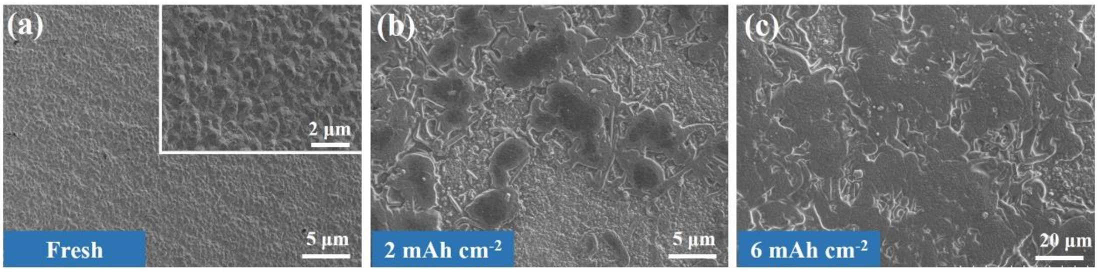


**Fig. S30** Morphological evolution of bare Cu foil electrode at different deposition capacities of Li

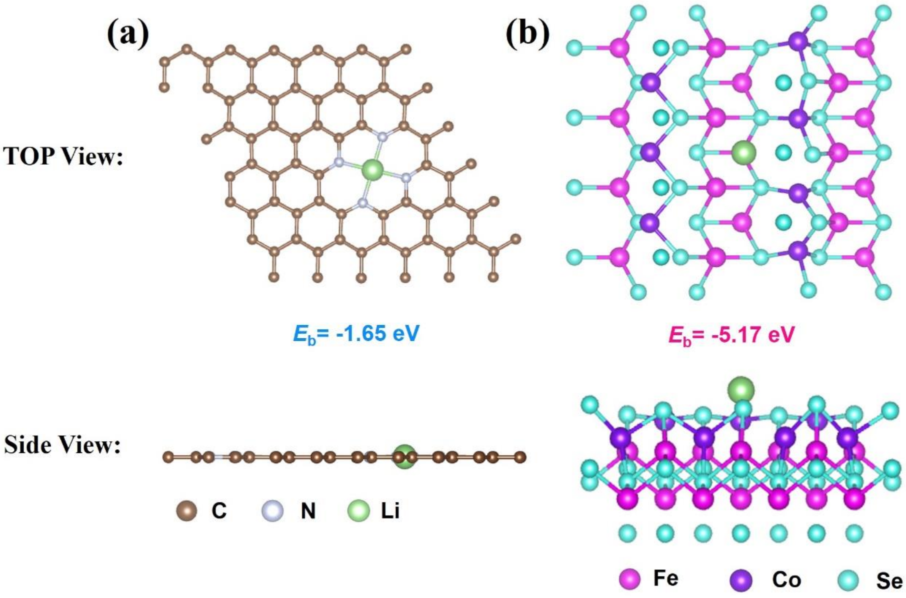


**Fig. S31** The optimized geometry conformation models and corresponding binding energies ($E_b$) of Li atom adsorbed on (**a**) N-doped carbon and (**b**) $Fe_2CoSe_4$

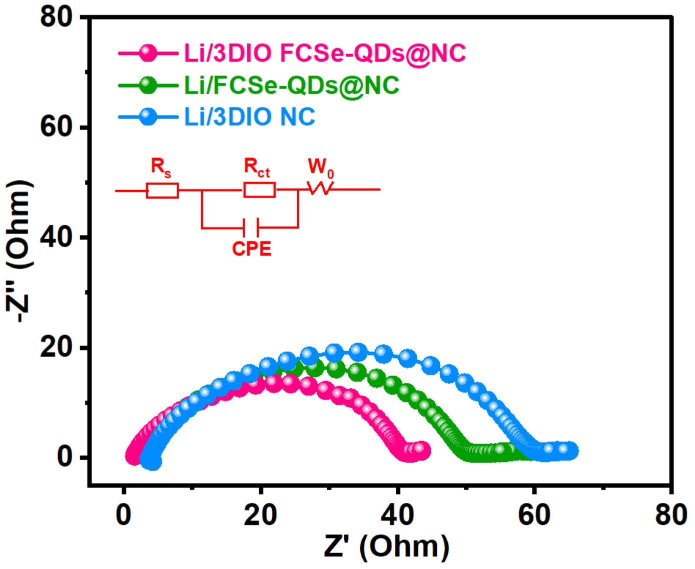


**Fig. S32** The corresponding Nyquist plots obtained after cycling at 1 mA cm$^{-2}$ with a plating/striping capacity of 1 mAh cm$^{-2}$

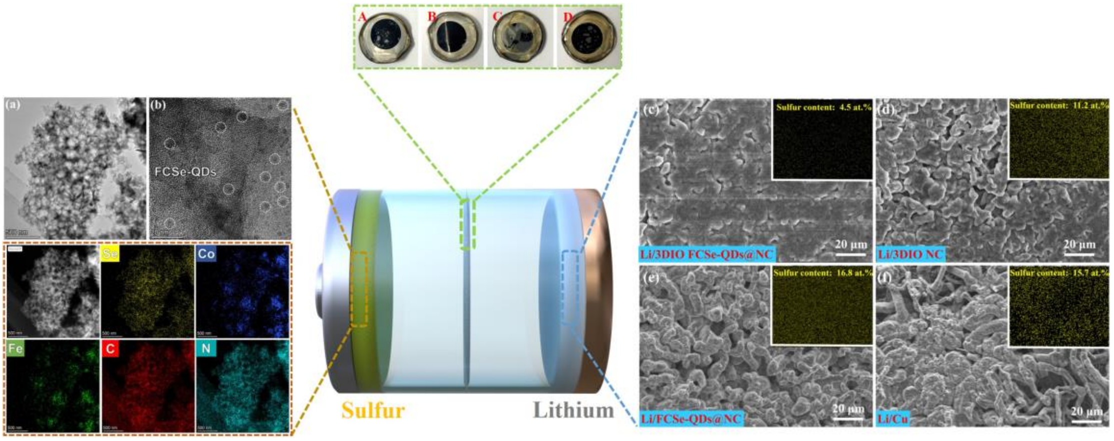


**Fig. S33** TEM images and corresponding EDS mappings of cathode and anode from the disassembled 3DIO FCSe-QDs@NC-based full cell after cycling for 2000 cycles at 2C

**Note:** As shown in Fig. S33, the 3DIO FCSe-QDs@NC host recovered from the disassembled cell still exhibits 3D-ordered porous morphology without obvious structural damage, indicating good mechanical stability. Importantly, one can clearly observe that the FCSe-QDs still maintain a highly dispersed distribution on the carbon skeleton without obvious agglomeration or disappearance. And the uniform distribution of Se, Fe, and Co elements further confirms the excellent stability of QDs during cycling. And, the separators from the disassembled 3DIO FCSe-QDs@NC-based full cells (S/3DIO FCSe-QDs@NC||Li/3DIO FCSe-QDs@NC and S/3DIO FCSe-QDs@NC||Li/Cu) present pale yellow color, while other two controls samples show dark yellow-brown color, which indicates that LiPSs diffusion is effectively blocked and only a small amount of LiPSs is dissolved into the electrolyte. Furthermore, the Li anode of the cycled cell displays relatively smooth and compact surface. And the low intensity of the EDS sulfur signal shows a negligible amount of LiPS reaching the anode.

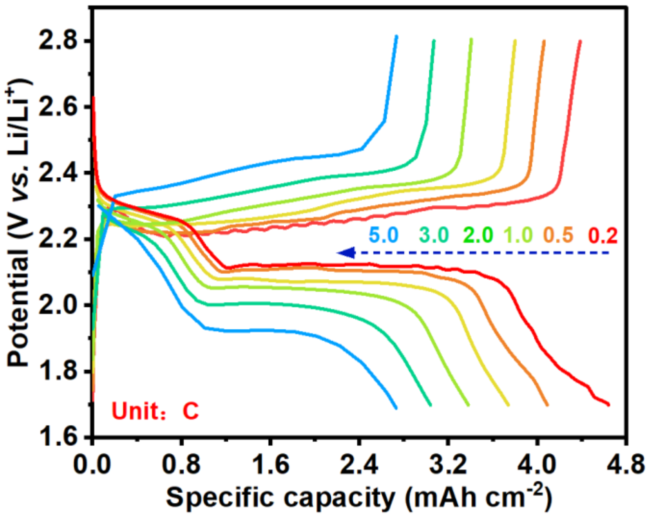


**Fig. S34** The discharge/charge profiles of the 3DIO FCSe-QDs@NC-based full cell at different current rates

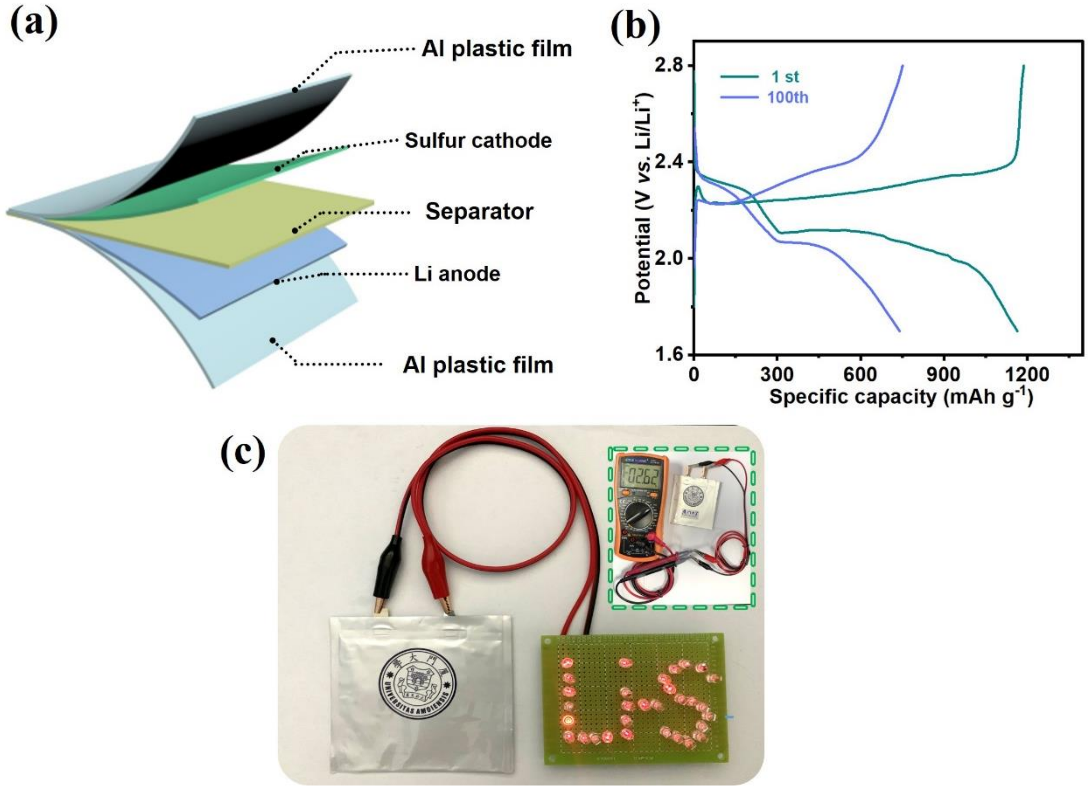


**Fig. 35** (**a**) Schematic diagram of pouch battery. (**b**) The discharge/charge profiles of the 3DIO FCSe-QDs@NC-based pouch cell at the first and last cycles. (**c**) Digital photographs of the LED device lit by pouch cell

**Table S1** Lithium-ion diffusion rates ($D_{Li^+}$, cm$^{-2}$ s$^{-1}$) of 3DIO FCSe-QDs@NCS batteries paired with different cathodes

| Sample | Peak I | Peak II | Peak III |
|---|---|---|---|
| **3DIO FCSe-QDs@NC** | 2.15×10$^{-7}$ | 3.92×10$^{-7}$ | 5.26×10$^{-7}$ |
| **FCSe-QDs@NC** | 2.05×10$^{-7}$ | 3.91×10$^{-7}$ | 5.18×10$^{-7}$ |
| **3DIO NC** | 1.85×10$^{-7}$ | 3.65×10$^{-7}$ | 4.89×10$^{-7}$ |

**Table S2** EIS fitting results of Li–S batteries paired with different cathodes before cycling

| Sample | $R_s$(Ω) | $R_{ct}$(Ω) |
|---|---|---|
| **3DIO FCSe-QDs@NC** | 11.8 | 52.2 |
| **FCSe-QDs@NC** | 20.5 | 75.1 |
| **3DIO NC** | 25.4 | 128.8 |

**Table S3** EIS fitting results of Li–S batteries paired with different cathodes after cycling

| Sample | $R_s$(Ω) | $R_p$(Ω) | $R_{ct}$(Ω) |
|---|---|---|---|
| **3DIO FCSe-QDs@NC** | 1.2 | 15.2 | 10.6 |
| **FCSe-QDs@NC** | 3.1 | 38.5 | 33.2 |
| **3DIO NC** | 5.2 | 48.7 | 42.1 |

**Table S4** EIS fitting results of Li||Li symmetrical cells based on different host matrices

| Sample | $R_s$(Ω) | $R_{ct}$(Ω) |
|---|---|---|
| **3DIO FCSe-QDs@NC** | 1.97 | 38.5 |
| **FCSe-QDs@NC** | 3.5 | 45.6 |
| **3DIO NC** | 4.1 | 51.1 |

**Table S5** Comparisons of the cycling performance of Li||Li symmetric cells with different hosts

| Li host | Current density [mA cm$^{-2}$] | Capacity [mAh cm$^{-2}$] | Cycle time [h] | Overpotential [mV] | Refs. |
|---|---|---|---|---|---|
| 3DIO FCSe-QDs@NC | 1 | 1 | 1400 | 13 | This work |
| | 3 | 3 | 800 | 28 | |
| | 5 | 5 | 500 | 65 | |
| HPTCF | 1 | 1 | 300 | 14 | [S16] |

| | | | | | |
|---|---|---|---|---|---|
| $Zn_1$-HNC | 3 | 3 | 400 | 42 | [S17] |
| $Ni_2Co$@rGO | 0.5 | 1 | 200 | 16.6 | [S18] |
| Co/N-PCNSs | 0.5 | 0.5 | 350 | 17 | [S19] |
| $WSe_2$/NG | 3 | 1 | 700 | 18 | [S9] |
| N/O-codoped | 2 | 2 | 500 | 40 | [S20] |
| MXene/COF | 1 | 1 | 400 | 25 | [S21] |
| N-doped graphene | 1 | 4 | 800 | 24 | [S22] |
| CNF@Ni | 0.5 | 1 | 1000 | 25 | [S23] |
| $Nb_4N_5$–$Nb_2O_5$ | 1 | 1 | 1000 | 10.5 | [S24] |
| TiN-VN@CNFs | 2 | 1 | 1000 | 24 | [S25] |

**Table S6** Comparison of the electrochemical performance based on S/3DIO FCSe-QDs@NC cathode with reported state-of-the-art works based on TMSes

| **Materials** | **Rate capability [mAh g$^{-1}$]** | **Capacity retention [mA g$^{-1}$]/Cycle numbers** | **High S loading performance [Capacity/Sulfur loading]** | **Refs.** |
|---|---|---|---|---|
| 3DIO FCSe-QDs@NC | 781/5 C | 1157/300/0.2 C<br>661/2000/2 C | 3.91/4.5/100 cycles<br>6.53/8.5/100 cycles | This work |
| $Ni_{0.1}Zn_{0.1}Co_{0.8}Se_2$ | 681/2 C | 495/400/1.0 C | 2.76/4.6/100 cycles | [S26] |
| CC/$NiCoSe_2$-NiO | 776/2 C | 610/1000/1 C | 2.12/3.5/250 cycles | [S27] |
| $Ti_3C_2/(NiCo)_{0.85}Se$ | 600/5 C | 320/2000/1 C | 5.3/6.4/80 cycles | [S28] |
| Co-$MoSe_2$/MXene | 759/5 C | 670/500/0.5 C | 5.33/8.7/200 cycles | [S29] |
| $VSe_2$ | 600/8 C | 782.2/500/1C | 4.04/6.1/200 | [S30] |
| RGO-$CoSe_2$ | 695.7/2C | 741.2/400/1 | 4.18/3.8/50 | [S31] |
| $C_2N$@$NbSe_2$ | 683/5 | 752.1/500/1 | 3.7/5.6/80 | [S32] |
| $MoSe_2$@rGO | 863/2 | 672/500/2 | 5.88/4.2/120 | [S33] |

| | | | | |
|---|---|---|---|---|
| CoSe@C | - | 715/600/1 | 5.8/6.2/100 | [S34] |
| CoZn-Se | 844/3 | 360/2000/2 | 6.6/7.8/30 | [S35] |
| CoSe | 754.3/3 | 414.3/1200/1 | 4.1/5.6/30 | [S6] |
| ZnSe | 743.2/6 | 743/200/1 | 3.6/3.2/120 | [S36] |
| $VSe_2$–VG@CC | 450/5 | 334/800/5 | 4.9/9.6/0.2 | [S37] |
| NG/$WSe_2$ | 570/5 | 750/500/1 | 3.4/5.2/350 | [S38] |

**Table S7** Comparison of the electrochemical performance based on 3DIO FCSe-QDs@NC with previously reported state-of-the-art full-cell works

| **Dual functional host** | **Rate capability [mAh g$^{-1}$]** | **Capacity retention [mA g$^{-1}$]/Cycle numbers** | **High S loading performance [Capacity/Sulfur loading]** | **Electrolyte/ sulfur ratio** | **Refs.** |
|---|---|---|---|---|---|
| 3DIO FCSe-QDs@NC | 745/5 C | 715/800/2.0 C | 3.91/4.5/100 cycles<br>6.53/8.5/100 cycles | 4.4<br>4.1 | This work |
| ZCNC@GC | 600/5 C | 849/200/0.5 C | 1.8/3.0/100 cycles<br>2.67/5.0/100 cycles | 10 | [S39] |
| NbC/Co⊂N-PCFs | 704/5 C | 783/500/0.3 C | 6.1/6.7/ 50 cycles | 10 | [S40] |
| CoTe⊂NCGs | 692/5 C | 513/800/2.0 C | 3.31/3.8/50 cycles | 4.2 | [S41] |
| TiN-3VN@CNFs | 650/5 C | 576/600/2.0 C | 5.5/5.6/100 cycles | 15 | [S25] |
| CoSe@C | - | 715/600/ 1.0 C | 5.8/6.2/100 cycles | 4.5 | [S34] |
| Hollow $TiO_2$-TiN | 564.7/4 C | 639/500/2.0 C | 1.8/3.0/100 cycles | 15 | [S42] |
| $Ni_2Co$@rGO | 590/2 C | 600/300/0.5 C | 3.5/4.0/100 cycles | 6.0 | [S18] |
| $Co_4N$/WCP | 841/2 C | 807/500/0.5 C | 3.36/4.0/150 cycles | - | [S43] |
| 3DRGO/NC | 450/5 C | 550/400/0.5 C | - | - | [S44] |

## Supplementary References

[S1] G. Kresse, J. Furthmüller, Efficient iterative schemes for ab initio total-energy calculations using a plane-wave basis set. Phys. Rev. B **54**(5), 11169 (1996). https://doi.org/10.1103/PhysRevB.54.11169

[S2] G. Kresse, D. Joubert From ultrasoft pseudopotentials to the projector

augmented-wave method. Phys. Rev. B **59**(25), 1758 (1999). https://doi.org/10.1103/PhysRevB.59.1758

[S3] S. Grimme, J. Antony, S. Ehrlich, H. Krieg, A consistent and accurate ab initio parametrization of density functional dispersion correction (DFT-D) for the 94 elements H-Pu. J. Chem. Phys. **132**(15), 154104 (2010). https://doi.org/10.1063/1.3382344

[S4] G. Wang, L. Peng, K. Li, L. Zhu, J. Zhou et al., ALKEMIE: An intelligent computational platform for accelerating materials discovery and design. COMP Mater Sci. **186**(55), 110064 (2021). https://doi.org/10.1016/j.commatsci.2020.110064

[S5] W. Yao, W. Zheng, J. Xu, C. Tian, K. Han et al., ZnS-SnS@NC heterostructure as robust lithiophilicity and sulfiphilicity mediator toward high-rate and long-life lithium-sulfur batteries. ACS Nano **15**(4), 7114-7130 (2021). https://doi.org/10.1021/acsnano.1c00270

[S6] Z. Ye, Y. Jiang, L. Li, F. Wu, R. Chen, A high-efficiency CoSe electrocatalyst with hierarchical porous polyhedron nanoarchitecture for accelerating polysulfides conversion in Li-S batteries. Adv. Mater. **32**(32), 2002168 (2020). https://doi.org/10.1002/adma.202002168

[S7] J. Pu, W. Gong, Z. Shen, L. Wang, Y. Yao et al., $CoNiO_2/Co_4N$ heterostructure nanowires assisted polysulfide reaction kinetics for improved lithium-sulfur batteries. Adv. Sci. **9**(4), 2104375 (2022). https://doi.org/10.1002/advs.202104375

[S8] J. Qian, Y. Xing, Y. Yang, Y. Li, K. Yu et al., Enhanced electrochemical kinetics with highly dispersed conductive and electrocatalytic mediators for lithium-sulfur batteries. Adv. Mater. **33**(25), 2100810 (2021). https://doi.org/10.1002/adma.202100810

[S9] P. Wang, F. Sun, S. Xiong, Z. Zhang, B. Duan et al., $WSe_2$ flakelets on n-doped graphene for accelerating polysulfide redox and regulating li plating. Angew. Chem., Int. Ed. **61**(7), 202116048 (2022). https://doi.org/10.1002/anie.202116048

[S10] J. Cai, Z. Sun, W. Cai, N. Wei, Y. Fan et al., A robust ternary heterostructured electrocatalyst with conformal graphene chainmail for expediting bi-directional sulfur redox in Li–S batteries. Adv. Funct. Mater. **31**(23), 2100586 (2021). https://doi.org/10.1002/adfm.202100586

[S11] Z. Ali, M. Asif, X. Huang, T. Tang, Y. Hou, Hierarchically porous $Fe_2CoSe_4$ binary-metal selenide for extraordinary rate performance and durable anode of sodium-ion batteries. Adv. Mater. 1802745 (2018). https://doi.org/10.1002/adma.201802745.

[S12] H. Hu, J. Zhang, B. Guan, X.W. Lou, Unusual formation of CoSe@carbon nanoboxes, which have an inhomogeneous shell, for efficient lithium storage. Angew. Chem. Int. Ed. **55**(33), 9514-8 (2016). https://doi.org/10.1002/anie.201603852

[S13] Y. Ma, L. Wei, Y. Gu, L. Zhao, Y. Jing et al., Insulative ion-conducting lithium selenide as the artificial solid-electrolyte interface enabling heavy-duty lithium metal operations. Nano Lett. **21**(17), 7354-7362 (2021). https://doi.org/10.1021/acs.nanolett.1c02658

[S14] F. Liu, L. Wang, Z. Zhang, P. Shi, Y. Feng et al., A mixed lithium-ion

conductive $Li_2S/Li_2Se$ protection layer for stable lithium metal anode. Adv. Funct. Mater. **30**(23), 1903937 (2020). https://doi.org/ARTN 200160710.1002/adfm.202001607

[S15] L. Lin, F. Liu, Y. Zhang, C. Ke, H. Zheng et al., Adjustable mixed conductive interphase for dendrite-free lithium metal batteries. ACS Nano **16**(8), 13101-13110 (2022). https://doi.org/10.1021/acsnano.2c05832

[S16] W.L. Cai, G.R. Li, D. Luo, G.N. Xiao, S.S. Zhu et al., The dual-play of 3D conductive scaffold embedded with Co, N codoped hollow polyhedra toward high-performance Li-S Full Cell. Adv. Energy Mater. **8**(34), 1802561 (2018). https://doi.org/10.1002/aenm.201802561

[S17] H.D. Shi, X.M. Ren, J.M. Lu, C. Dong, J. Liu et al., Dual-functional atomic zinc decorated hollow carbon nanoreactors for kinetically accelerated polysulfides conversion and dendrite free lithium sulfur batteries. Adv. Energy Mater. **10**(39), 2002271 (2020). https://doi.org/10.1002/aenm.202002271

[S18] G. Li, W. Qiu, W. Gao, Y. Zhu, X. Zhang et al., Finely-dispersed $Ni_2Co$ nanoalloys on flower-like graphene microassembly empowering a bi-service matrix for superior lithium–sulfur electrochemistry. Adv. Funct. Mater. **32**(32), 2202853 (2022). https://doi.org/10.1002/adfm.202202853

[S19] S. Liu, J. Li, X. Yan, Q. Su, Y. Lu et al., Superhierarchical cobalt-embedded nitrogen-doped porous carbon nanosheets as two-in-one hosts for high-performance lithium-sulfur batteries. Adv. Mater. **30**(12), 1706895 (2018). https://doi.org/10.1002/adma.201706895

[S20] Y. Xie, H. Zhang, J. Yu, Z. Liu, S. Zhang et al., A novel dendrite-free lithium metal anode via oxygen and boron codoped honeycomb carbon skeleton. Small **18**(11), 2104876 (2022). https://doi.org/10.1002/smll.202104876

[S21] C. Wei, Y. Wang, Y. Zhang, L. Tan, Y. Qian et al., Flexible and stable 3D lithium metal anodes based on self-standing MXene/COF frameworks for high-performance lithium-sulfur batteries. Nano Res. **14**(10), 3576-3584 (2021). https://doi.org/10.1007/s12274-021-3433-9

[S22] Z. Zhao, J. Wang, M. Cheng, J. Wu, Q. Zhang et al., N-doped porous carbon-graphene cables synthesized for self-standing cathode and anode hosts of Li–S batteries. Electrochim. Acta **349**, 136231 (2020). https://doi.org/10.1016/j.electacta.2020.136231

[S23] Z. Zeng, W. Li, X. Chen, X. Liu, Bifunctional 3D hierarchical hairy foam toward ultrastable lithium/sulfur electrochemistry. Adv. Funct. Mater. **30**(52), 2004650 (2020). https://doi.org/10.1002/adfm.202004650

[S24] H. Shi, J. Qin, P. Lu, C. Dong, J. He et al., Interfacial engineering of bifunctional niobium (v)-based heterostructure nanosheet toward high efficiency lean-electrolyte lithium–sulfur full batteries. Adv. Funct. Mater. **31**(28), 2102314 (2021). https://doi.org/10.1002/adfm.202102314

[S25] Y. Yao, H. Wang, H. Yang, S. Zeng, R. Xu et al., A dual-functional conductive framework embedded with tin-vn heterostructures for highly efficient polysulfide and lithium regulation toward stable Li-S full batteries. Adv. Mater. **32**(6), 1905658 (2020). https://doi.org/10.1002/adma.201905658

[S26] L. Chen, Y. Xu, G. Cao, H.M.K. Sari, R. Duan et al., Bifunctional catalytic effect of $CoSe_2$ for lithium–sulfur batteries: single doping versus dual doping. Adv. Funct. Mater. **32**(8), 2107838 (2021). https://doi.org/10.1002/adfm.202107838

[S27] T. Li, K. Liu, S. Wang, Z. Liu, G. Liao et al., Mesoporous hierarchical $NiCoSe_2$-NiO composite self-supported on carbon nanoarrays as synergistic electrocatalyst for flexible lithium-sulfur batteries. J. Colloid Interface Sci. **629**(58), 114-124 (2022). https://doi.org/10.1016/j.jcis.2022.07.106

[S28] Y. Ren, S. Chang, L. Hu, B. Wang, D. Sun et al., A bidirectional electrocatalyst for enhancing $Li_2S$ nucleation and decomposition kinetics in lithium–sulfur batteries. J. Mater. Chem. A **10**(34), 17532-17543 (2022). https://doi.org/10.1039/d2ta05046c

[S29] W. Wang, L. Huai, S. Wu, J. Shan, J. Zhu et al., Ultrahigh-volumetric-energy-density lithium-sulfur batteries with lean electrolyte enabled by cobalt-doped $MoSe_2/Ti_3C_2T_x$ MXene bifunctional catalyst. ACS Nano **15**(7), 11619-11633 (2021). https://doi.org/10.1021/acsnano.1c02047

[S30] W. Tian, B. Xi, Y. Gu, Q. Fu, Z. Feng et al., Bonding $VSe_2$ ultrafine nanocrystals on graphene toward advanced lithium-sulfur batteries. Nano Res. **13**(10), 2673-2682 (2020). https://doi.org/10.1007/s12274-020-2909-3

[S31] L. Chen, W.W. Yang, J.G. Liu, Y. Zhou Decorating $CoSe_2$ hollow nanospheres on reduced graphene oxide as advanced sulfur host material for performance enhanced lithium-sulfur batteries. Nano Res. **12**(11), 2743-2748 (2019). https://doi.org/10.1007/s12274-019-2508-3

[S32] D. Yang, Z. Liang, C. Zhang, J. Biendicho, M. Botifoll et al., $NbSe_2$ meets $C_2N$: a 2D-2D heterostructure catalysts as multifunctional polysulfide mediator in ultra-long-life lithium-sulfur batteries. Adv. Energy Mater. **11**(36), 2101250 (2021). https://doi.org/10.1002/aenm.202101250

[S33] W.Z. Tian, B. Xi, Z. Feng, H.B. Li, J.K. Feng et al., Sulfiphilic few-layered $MoSe_2$ nanoflakes decorated rGO as a highly efficient sulfur host for lithium-sulfur batteries. Adv. Energy Mater. **9**(36), 1901896 (2019). https://doi.org/10.1002/aenm.201901896

[S34] J. He, A. Manthiram 3D CoSe@C aerogel as a host for dendrite-free lithium-metal anode and efficient sulfur cathode in Li-S full cells. Adv. Energy Mater. **10**(41), 2002654 (2020). https://doi.org/10.1002/aenm.202002654

[S35] Z. Ye, Y. Jiang, L. Li, F. Wu, R. Chen Self-assembly of 0D-2D heterostructure electrocatalyst from MOF and MXene for boosted lithium polysulfide conversion reaction. Adv. Mater. **33**(33), 2101204 (2021). https://doi.org/10.1002/adma.202101204

[S36] D. Yang, C. Zhang, J. Biendicho, X. Han, Z. Liang et al., ZnSe/N-doped carbon nanoreactor with multiple adsorption sites for stable lithium-sulfur batteries. ACS Nano **14**(11), 15492-15504 (2020). https://doi.org/10.1021/acsnano.0c06112

[S37] H. Ci, J. Cai, H. Ma, Z. Shi, G. Cui et al., Defective $VSe_2$-graphene heterostructures enabling in situ electrocatalyst evolution for lithium-sulfur batteries. ACS Nano **14**(9), 11929-11938 (2020). https://doi.org/10.1021/acsnano.0c05030

[S38] C. Zhang, B. Fei, D. Yang, H. Zhan, J. Wang et al., Robust lithium–sulfur

batteries enabled by highly conductive wse$_2$-based superlattices with tunable interlayer space. Adv. Funct. Mater. **32**(24), 2201322 (2022). https://doi.org/10.1002/adfm.202201322

[S39] K. Zhu, J. Chen, C. Guo, H. Wang, H. Li et al., Hierarchically constructed ZnO/$Co_3O_4$ nanoheterostructures synergizing dendrite inhibition and polysulfide conversion in lithium–sulfur battery. ACS Mater. Lett. **4**(7), 1358-1367 (2022). https://doi.org/10.1021/acsmaterialslett.2c00266

[S40] Y. Wei, B. Wang, Y. Zhang, M. Zhang, Q. Wang et al., Rational design of multifunctional integrated host configuration with lithiophilicity-sulfiphilicity toward high-performance Li-S full batteries. Adv. Funct. Mater. **31**(3), 2006033 (2020). https://doi.org/10.1002/adfm.202006033

[S41] B. Li, P. Wang, B. Xi, N. Song, X. An et al., In-situ embedding CoTe catalyst into 1D–2D nitrogen-doped carbon to didirectionally regulate lithium-sulfur batteries. Nano Res. **15**(10), 8972-8982 (2022). https://doi.org/10.1007/s12274-022-4537-6

[S42] P. Xue, K. Zhu, W. Gong, J. Pu, X. Li et al., “One Stone Two Birds” design for dual-functional $TiO_2$-TiN heterostructures enabled dendrite-free and kinetics-enhanced lithium-sulfur batteries. Adv. Energy Mater. **12**(18), 2200308 (2022). https://doi.org/10.1002/aenm.202200308

[S43] W. Zhang, B. Xu, L. Zhang, W. Li, S. Li et al., $Co_4N$-decorated 3D wood-derived carbon host enables enhanced cathodic electrocatalysis and homogeneous lithium deposition for lithium-sulfur full cells. Small **10**(6), 2105664 (2021). https://doi.org/10.1002/smll.202105664

[S44] R. Lu, M. Cheng, L. Mao, M. Zhang, H. Yuan et al., Nitrogen-doped nanoarray-modified 3D hierarchical graphene as a cofunction host for high-performance flexible Li-S battery. EcoMat **2**(1), 12010 (2020). https://doi.org/10.1002/eom2.12010
